# The Complex Relationship Between HDL/LDL Cholesterol, Stroke and Cardiovascular Disease

**DOI:** 10.3390/healthcare14101371

**Published:** 2026-05-17

**Authors:** Mark Parker, Tanja Novaković, Milica Krga Rastović, Vanesa Benković, Iñaki Gutierrez-Ibarluzea

**Affiliations:** 1 ZEM Solutions, 11050 Belgrade, Serbia; milica@zem-solutions.com; 2 School of Public Health, Faculty of Medicine, University of Zagreb, 10000 Zagreb, Croatia; vanesa.benkovic@farmakoekonomika.hr; 3 OSTEBA—Basque Office for Health Technology Assessment, Health Research and Innovation Directorate, Ministry for Health, Basque Government, 01010 Vitoria-Gasteiz, Spain; i-gutierrezibarluzea@euskadi.eus

**Keywords:** big data analytics, multi-disease modelling, cardiovascular risk modelling, LDL-C, HDL-C, ASCVD, myocardial infarction, stroke

## Abstract

**Background and Aims**: Atherosclerotic cardiovascular disease (ASCVD) remains a leading cause of mortality worldwide, with lipid abnormalities playing a central role in disease development. While the causal role of low-density lipoprotein cholesterol (LDL-C) in ASCVD is well-established, the long-term population impact of combined lipid profiles, particularly the HDL-C/LDL-C ratio, remains less clearly quantified. This study aimed to estimate the lifetime burden of cardiovascular outcomes associated with different lipid risk profiles using a patient-level simulation framework. **Methods:** The authors analyzed projected lifetime ASCVD events across four HDL-C/LDL-C risk strata, ranging from low (≥0.45) to very high (<0.25), using the National Health Model Database of Projected and Estimated Outcomes (NHM-DPEO)—a digital twin of national healthcare systems built from multiple data sources, including national health and demographic statistics and estimates from the relevant literature. The framework is structured as a patient-level simulation model that projects individual health trajectories over a lifetime horizon. Model outputs were assessed for plausibility by comparison with published epidemiological estimates. **Results:** The NHM simulation revealed a strong, graded relationship between lipid profiles and cardiovascular survival. Life expectancy declined from 80.2 years in the lowest risk group (HDL-C/LDL-C ≥ 0.45) to 63.0 years in the very-high-risk group (HDL-C/LDL-C < 0.25), a reduction of 17.2 years, with 13.7 fewer QALYs. Similarly, participants with LDL-C > 5.0 mmol/L had a life expectancy 13.4 years shorter than those with LDL-C < 3.1 mmol/L. The burden of ASCVD increased exponentially with worsening lipid ratios: MI events rose from 5000 to 73,090 per 100,000 births, with onset in the highest risk group occurring as early as age 20. Ischaemic heart disease followed a similar pattern, showing up to 92% of events attributable to elevated lipid risk. While ischaemic stroke risk displayed a more complex pattern due to earlier MI mortality in high-risk groups, overall cardiovascular mortality and lifetime event burden were dominated by LDL-driven disease. These findings demonstrate that sustained LDL-C reduction and balanced HDL-C/LDL-C ratios confer substantial survival benefits across both sexes and all age groups. **Conclusions:** This study shows that lipid balance has a decisive influence on cardiovascular survival. Sustained LDL-C reduction and favourable HDL-C/LDL-C ratios markedly extend life expectancy and delay the onset of MI and IHD. The magnitude of this survival benefit highlights the need for early and continuous lipid control as a cornerstone of ASCVD prevention. The NHM quantifies these lifetime effects, offering valuable insights for targeted strategies that improve both longevity and quality of life.

## 1. Introduction

Cardiovascular diseases (CVDs) are a major cause of death in Europe with more than 60 million potential years of life lost [1]. CVDs present major health challenges and are a cause of health disparities [2]. Among all CVDs, ischaemic heart disease (IHD) and ischaemic stroke (IS) lead to the highest number of deaths globally [3]. Atherosclerosis, the underlying pathological process in most cardiovascular diseases, is characterized by the accumulation of cholesterol-rich lipoproteins within the arterial wall, leading to plaque formation, vascular inflammation, and eventual thrombotic events [4].

Despite the many risk factors that have been elucidated as contributors to the onset of atherosclerotic CVD, and despite advances in preventive cardiology, the long-term population impact of lipid exposure remains difficult to quantify [5,6,7,8].

After the evaluation of evidence from genetic studies, prospective epidemiologic cohort studies, Mendelian randomisation studies, and randomized trials of LDL-lowering therapies, the European Atherosclerosis Society Consensus Panel in 2017 published a consensus statement which concludes that low-density lipoprotein cholesterol (LDL-C) is the cause of atherosclerotic cardiovascular disease [5]. Given the implied high burden of increased LDL-C globally, there is a great need of estimating the long-term health and economic consequences of it [9].

Health system modelling and patient-level simulation frameworks are increasingly used to estimate the long-term impact of cardiovascular risk factors and interventions. Digital twin approaches enable the integration of epidemiological evidence, demographic structure, and disease progression pathways in order to project lifetime disease burden and healthcare outcomes. Such methods have been increasingly applied in cardiovascular and metabolic disease research to evaluate long-term risk trajectories and their implications for prevention strategies [10,11,12].

**Figure 1:**
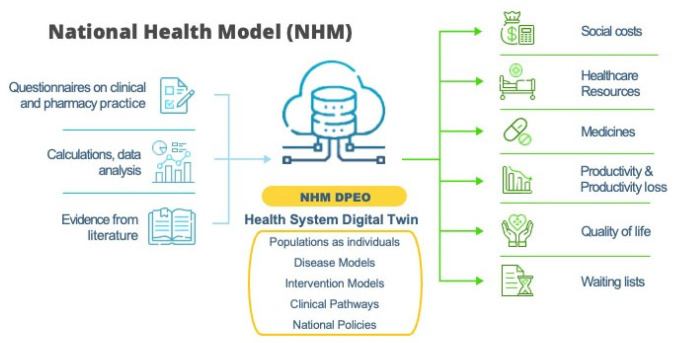
National Health Model overview scheme. Abbreviations: NHM—National Health Model; DPEO—Database of Projected and Estimated Outcomes.

The National Health Model (NHM), developed by ZEM Solutions, is a patient-level multi-disease simulation framework designed to model long-term health outcomes within national healthcare systems [13]. The model integrates epidemiological data, demographic structures, disease risk equations, and treatment pathways to simulate individual health trajectories over the lifetime horizon. By combining evidence from multiple data sources, NHM computes estimates of disease incidence, mortality, healthcare utilization, and quality-adjusted life years (QALYs) across populations (Figure 1).

The NHM Database of Projected and Estimated Outcomes (NHM-DPEO) incorporates evidence on disease natural history, healthcare service utilization, and treatment outcomes, allowing the modelling of interactions between multiple chronic diseases and cardiovascular risk factors. Recent advances in data analysis have enabled the efficient analysis of the large datasets generated by digital twin simulations on consumer hardware.

Although LDL-C is the principal causal lipid determinant of ASCVD, cardiovascular risk is not determined by LDL-C alone. HDL-C has long been inversely associated with cardiovascular risk in observational studies, even though its causal role remains less certain than that of LDL-C. In this context, the HDL-C/LDL-C ratio may serve as an integrated marker of lipid imbalance, reflecting the combined contribution of atherogenic and protective lipoprotein fractions to cardiovascular risk. Epidemiological studies have linked this ratio to myocardial infarction, stroke, and all-cause mortality, suggesting that combined lipid imbalance may provide a more informative measure of long-term risk than isolated lipid measures. However, the lifetime burden of cardiovascular events across HDL-C/LDL-C strata remains insufficiently quantified, particularly when competing mortality risks are taken into account.

The aim of this study was to quantify the long-term impact of lipid profiles on ASCVD outcomes, with a particular focus on the HDL-C/LDL-C ratio and lifetime disease burden.

## 2. Methods

All data and R code used in this study are included in the Supplementary Materials.

NHM facilitates the integration of multiple disease and economic models from different sources. Studies examining diabetes such as UKPDS 68 [14,15] and UKPDS 60 [16] have long shown HDL-C- and LDL-C-associated risks in several diseases for diabetic populations. A recent study on the UK biobank data looked further into these relationships in a general population [17]. These risk functions were used in a large, multi-disease discrete event simulation on the NHM to estimate the mortality and morbidity of ASCVD over the lifetime of a population based on the level of lipids risk. Model outputs were evaluated for plausibility by comparing projected event rates and life expectancy patterns with published epidemiological studies.

LDL-C, HDL-C and triglycerides levels and progression were projected per person, utilizing CDC lipid distribution data stratified by age and sex. Although the primary analysis focuses on the HDL-C/LDL-C ratio, triglyceride levels were included in the simulation to maintain realistic lipid profile distributions and to capture their role within the broader metabolic risk environment influencing cardiovascular outcomes. Triglycerides represent an important risk factor in the development of cardiovascular diseases and are also correlated with HDL and LDL levels [18].

Following this, the number of events was projected for four risk groups according to the HDL-C/LDL-C ratio (Supplementary Materials, Table S1):

Low-risk HDL-C/LDL-C ≥ 0.45;
Moderate-risk HDL-C/LDL-C 0.35–0.45;
High-risk HDL-C/LDL-C 0.25–0.35;
Very-high-risk HDL-C/LDL-C ≤ 0.25.

The HDL-C/LDL-C ratio categories were selected to represent progressively worsening combined lipid profiles. Because the analysis was stratified by ratio rather than by LDL-C alone, the very-high-risk group could include individuals with only moderately elevated LDL-C but markedly reduced HDL-C. This reflects combined lipid imbalance rather than isolated LDL-C elevation.

Ference et al. [19] proposed a hypothesis that cumulative LDL-C exposure or total atherosclerotic plaque burden, (defined as the total amount of LDL-C accumulated over the years of life) is a predictor of myocardial infarction (MI) risk. Once the cumulative LDL-C exposure threshold (5000 mg-Years) is reached, the cumulative risk of MI begins to rise exponentially.

Recently, Ference and authors extended LDL-C risk (Supplementary Materials, Table S2) to a definition of ASCVD including the first occurrence of fatal or non-fatal MI, fatal or non-fatal IS, coronary revascularization, or cardiovascular death [20].

Stroke risk reduction has also been estimated from a meta-analysis of 26 randomized controlled trials, which concluded that 1 mmol/L LDL-C reduction leads to 16% IS risk reduction [21].

Demographic and biometric distributions, including height, weight, and body mass index (BMI), were drawn from NHM-DPEO and validated against published population distributions from the CDC dataset. The population-level summary statistics by age and sex are shown in the Supplementary Materials, Table S3, and the BMI distribution curves by risk group and sex are shown in the Supplementary Materials, Figures S1–S6.

The population structure by age and sex across risk groups is visualized using population pyramids in the Supplementary Materials, Figures S7–S10. This helps to contextualize disease burden projections across age–sex cohorts within each lipid risk category.

Additional cardiovascular risk factors were incorporated into the model using embedded prevalence and disease progression modules. Smoking prevalence was stratified by age and sex, as illustrated in the Supplementary Materials, Figure S11. Diabetes prevalence, which has a strong interaction with lipid metabolism and stroke risk, was adjusted by both age and sex (Supplementary Materials, Figure S12). Glycated hemoglobin (HbA1c) levels were also modelled by sex and HDL-C/LDL-C risk group to capture the broader metabolic risk burden, with the results shown in the Supplementary Materials, Figures S13 and S14. Hypertension and atrial fibrillation were included as age- and risk-specific modifiers, utilizing validated ICD-10 diagnostic codes (I10 for hypertension; I48 for atrial fibrillation); their prevalence is presented in the Supplementary Materials, Figures S15–S18. Lastly, systolic blood pressure (SBP), a key determinant of both ASCVD and stroke, was modelled by sex and lipid risk group, as shown in the Supplementary Materials, Figures S19 and S20.

The simulation projected the full spectrum of ASCVD outcomes over the lifetime of individuals, accounting for the influence of lipid risk strata and comorbidities. Specifically, the model estimated the incidence, timing, and cumulative burden of MI, IHD, IS, and heart failure (HF), in those both with and without a prior history of IHD. In addition, it captured cardiovascular and non-cardiovascular mortality events. These outcomes were dynamically modelled over time based on age, sex, lipid profiles, and interactions with other risk factors such as diabetes and hypertension. Cause-specific mortality patterns were summarized and ranked by risk group in the Supplementary Materials, Table S4, providing a comprehensive overview of leading fatal outcomes associated with each lipid risk category. The trajectory and burden of these cardiovascular events were visualized using cumulative incidence analyses, enabling direct comparisons between groups and enhancing the interpretability of disease progression across the lifespan.

Health-related quality of life (HRQoL) was comprehensively modelled within the NHM to assess the long-term impact of lipid-related ASCVD risks. The QALY profiles, stratified by gender and HDL-C/LDL-C risk groups, are presented in the Supplementary Materials, Figures S21 (males) and S22 (females). These figures illustrate how HRQoL declines with increasing lipid risk, particularly in the very-high-risk group (HDL-C/LDL-C < 0.25), where reduced life expectancy and higher ASCVD event rates contribute to significantly lower QALYs. The profiles provide valuable insights into the differential impact of lipid profiles on long-term health outcomes across genders, highlighting the compounded burden of comorbidities such as diabetes and hypertension in higher risk groups.

## 3. Results

NHM projections showed a strong graded association between lipid risk and both life expectancy and health-related quality of life (HRQoL). Individuals in the highest risk group were estimated to live 17.21 years less than those in the lowest risk group, with a reduction of 13.67 lifetime QALYs.

The very-high-risk group (HDL-C/LDL-C < 0.25) showed a lower mean LDL-C level than some of the intermediate-risk groups (Table 1 and Table 2). This reflects the ratio-based stratification used in the analysis, in which very low HDL-C levels can define the highest risk group despite only moderately elevated LDL-C values. The same pattern was observed in both sexes, with males showing more pronounced absolute differences.

**Figure 2:**
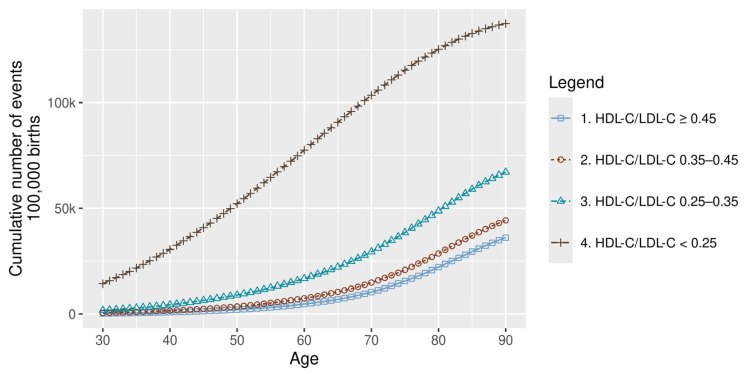
Cumulative ASCVD events by risk group. ASCVD: atherosclerotic cardiovascular disease; HDL-C: high-density lipoprotein cholesterol; and LDL-C: low-density lipoprotein cholesterol.

As for the ASCVD, the cumulative burden of ASCVD events—comprising fatal and non-fatal HF, IHD, MI, and IS—escalated markedly with worsening lipid profiles. An exponential increase in event rates was observed with declining HDL-C/LDL-C ratios (Figure 2).

Myocardial infarction showed the clearest gradient across lipid risk groups. The projected number of lifetime MI events by risk group ranged from 5000 for the lowest risk group to 73,090 for HDL-C/LDL-C < 0.25, with the event-specific mortality ranging from 49 to 64%.

Differences were evident not only in the cumulative burden, but also in terms of timing. In the highest risk group, the first MI events occurred as early as age 20, approximately two decades earlier than in the lowest risk group (Figure 2).

Across the life course, MI risk remained consistently higher in the HDL-C/LDL-C < 0.25 group, with the steepest increase observed between 50 and 75 years of age.

Overall, the projected number of lifetime MI events was substantially higher in the HDL-C/LDL-C < 0.25 group than in the HDL-C/LDL-C ≥ 0.45 group (See Table 1 and Table 3). By contrast, the lowest risk group had a projected lifetime MI risk of approximately 5% (Table 3).

Ischaemic heart disease showed a pattern broadly similar to that observed for myocardial infarction, but with a higher event-specific mortality range of 34% to 86% (Table 3). In the very-high-risk HDL-C/LDL-C group, the model attributed up to 92% of IHD events to elevated lipid risk over long exposure durations. Although IHD contributed substantially to cumulative disease burden and HRQoL loss, the associated loss of life years was lower than that observed for MI (Table 3).

**Figure 3:**
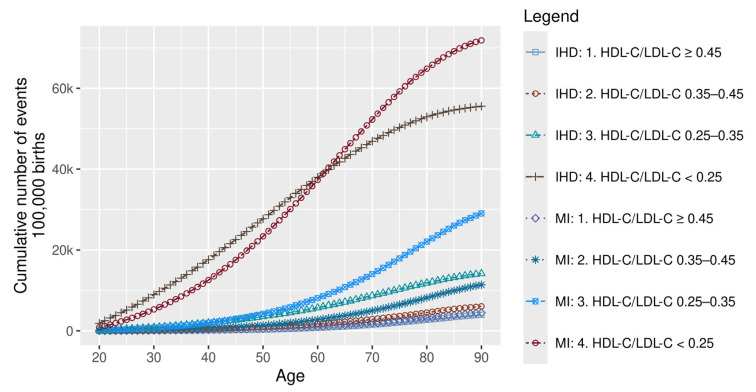
Cumulative MI and IHD events by risk group. HDL-C: high-density lipoprotein cholesterol; IHD: ischaemic heart disease; LDL-C: low-density lipoprotein cholesterol; and MI: myocardial infarction.

The cumulative lifetime event rates of MI and IHD by risk ratios are presented in Figure 3.

In the low- to high-risk groups, total and fatal MI events were almost twice as frequent in men as in women. This sex difference persisted in the very-high-risk group, although it was less articulated.

Heart failure incidence without prior ischaemic heart disease showed no consistent association with LDL-C, but remained elevated across higher HDL-C/LDL-C risk groups (Table 3).

**Figure 4:**
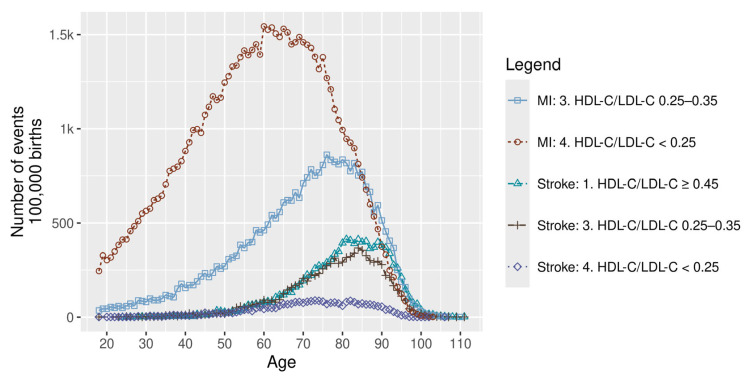
Selected incident non-fatal MI and IS events by age and risk group. HDL-C: high-density lipoprotein cholesterol; LDL-C: low-density lipoprotein cholesterol; and MI: myocardial infarction.

**Figure 5:**
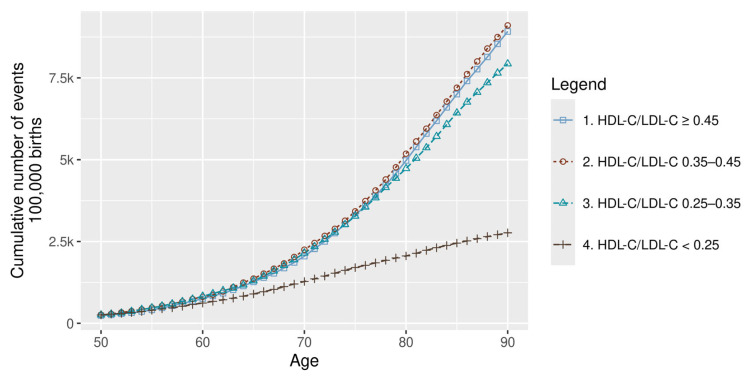
Cumulative stroke events by risk group. HDL-C: high-density lipoprotein cholesterol; LDL-C: low-density lipoprotein cholesterol.

**Figure 6:**
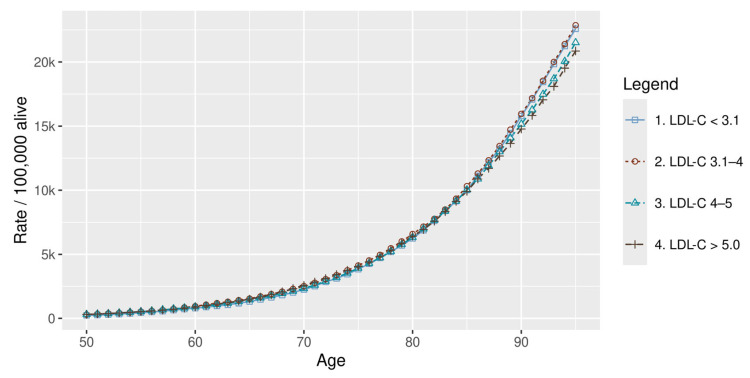
Mortality adjusted cumulative stroke risk by LDL-C. LDL-C: low-density lipoprotein cholesterol.

The pattern for ischaemic stroke (IS) differed from that observed for myocardial infarction and ischaemic heart disease. Lifetime non-fatal IS events were lower in the very-high-risk group than in the moderate-risk group (Figure 4). Across the full simulation, the very-high-risk group also experienced fewer total lifetime IS events than the moderate-risk group (Figure 5; Table 3). This pattern persisted after adjustment for differential mortality (Figure 6; Table 3).

## 4. Discussion

The present findings are broadly consistent with the established association between increased LDL-C levels and higher risks of myocardial infarction, ischaemic heart disease, and ischaemic stroke. However, when these relationships are examined over the lifetime horizon, their interaction with age, comorbidity burden, cumulative exposure, and competing mortality becomes more complex. These dynamics are not easily captured in conventional observational analyses and help explain why the lifetime cardiovascular burden may differ from instantaneous risk patterns. Previous studies have similarly highlighted the complexity of long-term cardiovascular risk modelling [19,22]. The projections in this study are based on previously published risk equations and epidemiological evidence describing the relationship between lipid levels and cardiovascular disease. Therefore, the model does not aim to generate new causal evidence regarding the role of LDL-C in ASCVD. Instead, the contribution of this study lies in integrating these established relationships within a multi-disease, patient-level simulation framework to quantify the cumulative lifetime impact of lipid profiles on cardiovascular outcomes. By modelling competing risks, disease progression, and long-term survival trajectories simultaneously, the NHM framework provides a comprehensive estimate of lifetime ASCVD burden across different HDL-C/LDL-C risk strata. Such lifetime projections are difficult to obtain from conventional cohort studies, which typically observe populations over shorter time horizons.

Atherosclerosis is a cumulative process driven by long-term exposure to atherogenic lipoproteins, which provides the biological basis for modelling lifetime lipid burden [23,24,25,26]. The progressive course of the disease is substantively validated by the comparison of our results with a recent study examining familial hypercholesterolemia (FH) [27]. The study showed that the life expectancy of genetically defined heterozygous FH is 69.3 years, which closely aligns with life expectancy of our study population when LDL-C > 4, providing an external plausibility check for the model outputs, presented in Table 2 (69.68 years).

Additionally, the projected lifetime MI risk in the lowest risk HDL-C/LDL-C group was approximately 5% (Table 3), corresponding to an estimated cumulative LDL-C exposure of 207.31 mmol-years based on the mean LDL-C level and life expectancy shown in Table 1. This closely aligns with previous estimates suggesting an approximately 4% MI risk at comparable cumulative LDL exposure levels [19].

LDL-C is a well-established causal driver of ASCVD, with each 1 mmol/L reduction associated with substantial reductions in major vascular events [22,28]. This well-established relationship provides the biological and epidemiological basis for interpreting the gradients observed across lipid risk groups in the present simulation.

LDL-C reduction remains a cornerstone of cardiovascular risk reduction, while higher HDL-C levels are generally associated with protective effects, although this relationship is not strictly linear. Epidemiological evidence indicates that higher HDL-C is linked to lower risks of myocardial infarction and ischaemic heart disease, particularly when LDL-C is well controlled [17]. However, a U-shaped association has been observed, with extremely high HDL-C levels (≥80 mg/dL) associated with increased all-cause and cardiovascular mortality, suggesting that HDL-C alone is not a sufficient therapeutic target [29]. In this context, the HDL-C/LDL-C ratio provides a more integrated measure of lipid-related risk. In the UK Biobank cohort, a low ratio (<0.4) was associated with significantly increased risks of myocardial infarction and ischaemic stroke [17]. Consistent with these findings, our results show that lower HDL-C/LDL-C ratios are associated with earlier onset and substantially higher lifetime burden of ASCVD events, while higher ratios correspond to greater longevity and lower cumulative risk. These findings support the concept that combined lipid imbalance, rather than isolated lipid measures, is a key determinant of long-term cardiovascular outcomes.

The relationship between LDL-C and stroke remains complex and has been debated in the literature. Meta-analyses of both observational studies and randomized trials consistently show that higher LDL-C levels are associated with increased ischaemic stroke risk and that LDL-C reduction reduces stroke incidence [30,31]. These findings support the established role of LDL-C in stroke pathophysiology, but do not capture the lifetime dynamics observed in simulation-based analyses. An observational study showed that LDL-C appears to be a less contributing factor to ASCVD in women than in men [32]. The projected number of MI events in male and female populations are coherent with the existing literature. A prospective population-based study included 471,998 participants without prior cardiovascular events. Over the course of seven years, 5081 participants experienced an MI, with an incidence three times higher in men than in women. This study showed that, in women, high blood pressure, smoking status and a history of diabetes result in a greater risk of MI than in men [33]. Life expectancy is higher in women regardless of lipid risk group, which is coherent with the epidemiological data on the longer lifespans of women [34].

Our findings indicate that myocardial infarction occurs substantially earlier in life than ischaemic stroke, with first events observed around age 20 versus 60, respectively.

Although LDL-C did not show a consistent direct association with heart failure incidence, the overall burden of heart failure remained elevated in higher-lipid-risk groups. This pattern suggests an indirect effect of lipid-related risk, mediated through comorbidity burden and prior cardiovascular events. Individuals in higher HDL-C/LDL-C risk categories exhibited a greater clustering of metabolic risk factors, including diabetes, hypertension, and atrial fibrillation, which likely contributed to the observed heart failure patterns.

Familial hypercholesterolemia is typically associated with approximately twofold higher LDL-C levels when left untreated [35]. Given the projections from our study, these patients are at a very high risk of MI and IS if left untreated. However, clinical evidence on the association between LDL-C and IS remains contradictory. Despite the elevated LDL-C, FH patients do not have a higher risk of IS, as recorded by a prospective matched cohort study on 5691 patients with genetically verified FH [36]. However, a large meta-analysis including 183,388 participants found that only clinical FH is related to a significantly higher risk of IS, yet genetically confirmed FH was not [37].

Unlike traditional observational analyses that assess instantaneous stroke risk, the present findings reflect lifetime accumulation of events under competing mortality risks, offering a different perspective on the relationship between lipid exposure and ischaemic stroke burden. In this context, an apparent inverse association was observed, whereby higher LDL-C levels were associated with a lower number of lifetime stroke events (Table 3; Figure 5). This pattern should not be interpreted as a protective biological effect. Rather, it reflects survivorship bias: individuals with higher lipid risk experience earlier mortality from myocardial infarction and ischaemic heart disease, reducing the likelihood of surviving to ages at which stroke incidence increases. Consequently, despite the higher instantaneous stroke risk in higher LDL-C groups, the cumulative number of lifetime stroke events remains lower, a pattern that persisted even after mortality adjustment (Figure 6). This effect was particularly pronounced in males, whereas in the very-high-risk female population, stroke events were more frequent, likely reflecting their longer survival (Table 3). Additional interactions with comorbidities such as diabetes may further influence lifetime stroke patterns, as individuals with lower lipid risk are more likely to survive to ages at which stroke risk becomes clinically relevant.

The implications for lipid-lowering therapies arise from the need to properly quantify both the short- and long-term health benefits of interventions targeting lipid levels. The impact on lifetime outcomes depends predominantly on their effect on atherosclerotic progression, as reflected in cumulative lipid exposure over time.

Existing risk estimates often treat disease probabilities under the assumption that lipid ratios are an instantaneous risk factor, rather than accumulated over time in the development of atherosclerosis.

Lipid levels tend to be stable with age, ref. [18] producing the results presented here; however, as our understanding of ASCVD has developed [5,19,20,26], this assumption is likely flawed when considering medications that modify LDL-C or HDL-C over a short period of time.

The value of the present analysis therefore lies not in identifying new biological associations, but in quantifying the long-term population impact of lipid exposure across the lifespan using an integrated simulation framework.

Several limitations of the present analysis should be acknowledged when interpreting these findings. Numerous studies highlight the protective role of regular physical activity in cardiovascular health [35,36]. Physical activity influences lipid metabolism by lowering LDL-C and increasing HDL-C levels, thereby reducing the risk of ASCVD and stroke. Socioeconomic status (SES) and psychosocial stress are also important determinants of cardiovascular outcomes, influencing health through multiple pathways including access to healthcare, lifestyle patterns, and metabolic regulation [38,39,40]. However, these factors—including physical activity, SES, psychosocial stress, and other lifestyle determinants such as nutritional habits—were not explicitly incorporated into the present model. The omission of these variables may influence the magnitude of projected outcomes. Future modelling efforts should aim to integrate lifestyle, socioeconomic, and psychosocial determinants in order to better capture the broader drivers of cardiovascular risk.

The simulation used in this study draws on datasets such as the UK Biobank and CDC data, which may not fully reflect the diversity of lipid metabolism across different ethnic groups and geographical regions. Cardiovascular risk profiles and responses to lifestyle or pharmacological interventions may vary substantially between populations, potentially limiting the generalisability of the findings.

In addition, the model assumes relatively stable lipid levels over time. In real-world clinical settings, lipid levels often fluctuate due to medication adherence, lifestyle changes, and other health interventions. A dynamic modelling framework that captures such variations could improve the accuracy of long-term cardiovascular risk projections. Although the long-term efficacy of lipid-lowering therapies such as statins is well-established, the model assumes consistent treatment adherence and effectiveness, which may not fully reflect real-world clinical practice.

A formal external validation of NHM projections against longitudinal cohort datasets was not performed in this study. However, several projected outcomes were compared with the epidemiological estimates reported in the literature. For example, the modelled life expectancy for individuals with high LDL-C levels closely aligns with estimates reported for patients with heterozygous familial hypercholesterolemia. Similarly, the projected lifetime myocardial infarction risk associated with cumulative LDL exposure is consistent with estimates reported in previous modelling studies. These comparisons provide indirect support for the plausibility of the model outputs. Nevertheless, future work should aim to formally validate NHM projections using real-world cohort data and national cardiovascular registries.

Another limitation is that the model does not differentiate between ischaemic and haemorrhagic stroke subtypes. While the present analysis focused primarily on ischaemic events, real-world datasets often include mixed stroke pathology. Because LDL-C and HDL-C may have different or even opposing associations with haemorrhagic stroke risk, aggregating stroke outcomes could obscure lipid-specific effects. Future modelling studies should therefore disaggregate stroke subtypes to provide more precise and clinically actionable estimates.

Further research should focus on the formal validation of NHM projections against longitudinal cohort data and national cardiovascular registries. In addition, future modelling efforts should incorporate broader determinants of cardiovascular risk, including lifestyle factors, socioeconomic status, and dynamic changes in lipid levels over time. Extending simulation frameworks in this way may improve the precision and generalisability of lifetime ASCVD projections.

## 5. Conclusions

This modelling analysis quantifies the projected lifetime impact of lipid profiles on cardiovascular risk within a population-based simulation framework. Higher LDL-C and lower HDL-C levels were associated with substantially increased projected risks of myocardial infarction and ischaemic heart disease, highlighting the importance of cumulative lipid exposure in shaping lifetime ASCVD burden. While elevated LDL-C also contributes to stroke risk, the lifetime distribution of stroke events reflects the interaction between lipid exposure, survival, and competing cardiovascular mortality. Individuals with favourable HDL-C/LDL-C ratios were projected to live longer and to experience delayed onset of atherosclerotic events.

These projections should be interpreted as model-based estimates derived from existing epidemiological evidence. From a public health perspective, sustained LDL-C reduction and early lipid management remain central strategies for reducing the long-term burden of ASCVD. Simulation frameworks such as NHM can support population-level forecasting and help inform preventive strategies aimed at improving both longevity and quality of life. These findings highlight the importance of adopting a lifetime perspective in cardiovascular risk assessment, where competing risks and cumulative exposure jointly shape observed disease patterns.

## Figures and Tables

**Table 1:** Life expectancy and QALYs by risk group.

Gender	Risk Group	Life Expectancy	Lifetime QALYs	Mean LDL-C (mmol/L)	Mean HDL-C (mmol/L)
All					
	1. HDL-C/LDL-C ≥ 0.45 (*n* = 100,000)	80.23	67.55	2.58	1.59
	2. HDL-C/LDL-C 0.35–0.45 (*n* = 100,000)	79.42	66.88	3.58	1.42
	3. HDL-C/LDL-C 0.25–0.35 (*n* = 100,000)	76.90	64.85	3.97	1.19
	4. HDL-C/LDL-C < 0.25 (*n* = 100,000)	63.02	53.87	3.50	0.76
Female					
	1. HDL-C/LDL-C ≥ 0.45 (*n* = 50,000)	82.02	68.22	2.63	1.68
	2. HDL-C/LDL-C 0.35–0.45 (*n* = 50,000)	81.46	67.76	3.73	1.49
	3. HDL-C/LDL-C 0.25–0.35 (*n* = 50,000)	79.64	66.31	4.02	1.21
	4. HDL-C/LDL-C < 0.25 (*n* = 50,000)	70.57	59.30	3.45	0.77
Male					
	1. HDL-C/LDL-C ≥ 0.45 (*n* = 50,000)	78.45	66.87	2.53	1.50
	2. HDL-C/LDL-C 0.35–0.45 (*n* = 50,000)	77.38	66.00	3.44	1.36
	3. HDL-C/LDL-C 0.25–0.35 (*n* = 50,000)	74.17	63.40	3.93	1.17
	4. HDL-C/LDL-C < 0.25 (*n* = 50,000)	55.48	48.45	3.54	0.74

HDL-C: high-density lipoprotein cholesterol; LDL-C: low-density lipoprotein cholesterol; and QALY: quality-adjusted life year.

**Table 2:** Life expectancy and QALYs by LDL-C group.

Gender	LDL Group	Life Expectancy	Lifetime QALYs
All			
	1. LDL-C < 3.1 (*n* = 100,000)	79.58	67.02
	2. LDL-C 3.1–4 (*n* = 100,000)	77.07	65.01
	LDL-C > 4 ^a^ (*n* = 200,000)	69.68	59.08
	3. LDL-C 4–5 (*n* = 100,000)	73.14	61.86
	4. LDL-C > 5.0 (*n* = 100,000)	66.23	56.29
Female			
	1. LDL-C < 3.1 (*n* = 50,000)	81.93	68.16
	2. LDL-C 3.1–4 (*n* = 50,000)	80.75	67.22
	3. LDL-C 4–5 (*n* = 50,000)	78.74	65.65
	4. LDL-C > 5.0 (*n* = 50,000)	74.08	62.01
Male			
	1. LDL-C < 3.1 (*n* = 50,000)	77.23	65.88
	2. LDL-C 3.1–4 (*n* = 50,000)	73.39	62.79
	3. LDL-C 4–5 (*n* = 50,000)	67.54	58.07
	4. LDL-C > 5.0 (*n* = 50,000)	58.38	50.57

LDL: low-density lipoprotein; LDL-C: low-density lipoprotein cholesterol; and QALY: quality-adjusted life year. ^a^ LDL-C > 4 is relevant for estimation of familial hypercholesterolemia life expectancy, see Discussion.

**Table 3:** Lifetime number of events/100,000 Births.

		HF ^a^		IHD ^b^		MI ^c^		Other ^d^	IS ^e^	
Gender	Risk Group	Total	Fatal	Total	Fatal	Total	Fatal	Fatal	Total	Fatal
All										
	1. HDL-C/LDL-C ≥ 0.45	13,960	1807	4370	3551	5000	2508	85,998	10,882	5192
	2. HDL-C/LDL-C 0.35–0.45	13,166	1958	6499	3746	12,642	6565	81,679	10,859	5180
	3. HDL-C/LDL-C 0.25–0.35	11,212	2298	14,686	5557	31,163	16,797	70,178	9031	4424
	4. HDL-C/LDL-C < 0.25	4623	1615	55,992	19,540	73,090	46,297	30,845	2925	1459
Female										
	1. HDL-C/LDL-C ≥ 0.45	15,356	1904	4580	3954	3082	1608	86,896	9320	4478
	2. HDL-C/LDL-C 0.35–0.45	14,682	2022	6258	4028	8256	4350	83,818	9710	4656
	3. HDL-C/LDL-C 0.25–0.35	13,128	2418	12,984	5448	21,820	11,590	75,112	8864	4384
	4. HDL-C/LDL-C < 0.25	7058	2366	47,218	16,008	61,046	37,872	41,220	4192	2104
Male										
	1. HDL-C/LDL-C ≥ 0.45	12,564	1710	4160	3148	6918	3408	85,100	12,444	5906
	2. HDL-C/LDL-C 0.35–0.45	11,650	1894	6740	3464	17,028	8780	79,540	12,008	5704
	3. HDL-C/LDL-C 0.25–0.35	9296	2178	16,388	5666	40,506	22,004	65,244	9198	4464
	4. HDL-C/LDL-C < 0.25	2188	864	64,766	23,072	85,134	54,722	20,470	1658	814

HDL-C: high-density lipoprotein cholesterol; HF: heart failure; IHD: ischaemic heart disease; IS: ischaemic stroke; LDL-C: low-density lipoprotein cholesterol; and MI: myocardial infarction. Notes: (^a^) HF = heart failure: first occurrence with no history of IHD (ICD10: I50); (^b^) IHD = Ischaemic Heart Disease (ICD10: I25); (^c^) MI = myocardial infarction (ICD10: I21, I22); (^d^) Other = deaths from other causes; and (^e^) IS = ischaemic stroke (ICD10: I63).

## Data Availability

The data presented in this study are openly available on GitHub account from the first corresponding author Mark Parker [link: https://github.com/mSparks43/healthcare-14-01371/ (accessed on 8 February 2026)].

## Supplementary Materials

The following supporting information can be downloaded at: https://www.mdpi.com/article/10.3390/healthcare14101371/s1, Table S1. Population at Risk by Risk Group; Table S2. Population at Risk by LDL Group; Table S3. Population heights and weights; Table S4. Top 10 causes of death by risk group; Figure S1. Female BMI, HDL-C/LDL-C ≥ 0.45; Figure S2. Female BMI, HDL-C/LDL-C 0.35 – 0.45; Figure S3. Female BMI, HDL-C/LDL-C < 0.35; Figure S4. Male BMI, HDL-C/LDL-C ≥ 0.45; Figure S5. Male BMI, HDL-C/LDL-C 0.35–0.45; Figure S6. Male BMI, HDL-C/LDL-C < 0.35; Figure S7. Low Risk Population pyramid; Figure S8. Moderate risk population pyramid; Figure S9. High Risk population pyramid; Figure S10. Very High Risk population pyramid; Figure S11. Smoking Prevalence; Figure S12. Diabetes Prevalence; Figure S13. Female HbA1c by Risk Group; Figure S14. Male HbA1c by Risk Group; Figure S15. Female Hypertension Prevalence by Risk Group; Figure S16. Male Hypertension Prevalence by Risk Group; Figure S17. Female Atrial Fibrilation Prevalence by Risk Group; Figure S18. Male Atrial Fibrilation Prevalence by Risk Group; Figure S19. Female SBP by Risk Group; Figure S20. Male SBP by Risk Group; Figure S21. Male QALY profiles; Figure S22. Female QALY profiles.
