# Supplementary material for: The Complex Relationship Between HDL/LDL Cholesterol, Stroke and Cardiovascular Disease
Source: Healthcare (Basel). 2026 May 17;14(10):1371. doi: 10.3390/healthcare14101371 (PMC13206512; doi:10.3390/healthcare14101371)
Supplement: Supplementary file 1 [file healthcare-14-01371-s001.zip › healthcare-4170270-supplementary.pdf]

Supplement 1, The Complex Relationship between HDL/LDL Cholesterol, Stroke and Cardiovascular Disease

Mark Parker, Tanja Novakovic, Milica Krga Rastović, Dr.sc. Vanesa Benkovic, Dr. Iñaki Gutierrez-Ibarluzea

Data Initialisation

```
library(NHMStandardLib)
library(flextable)


nhmSetPalette("bold")
folderInit()
options(scipen = 999)
setg11nLang("en")
if(params$rebuild){
  raw_data0 <- importNHMDataBase(
    "data"
    ,"_pop_20240827")

  message("got raw data")
  raw_data<-mapReduce_map_toFileList(raw_data0)
  mrEnv<-new.env()
  source("refSources/supplementalFuncs.R", local=mrEnv)
  gc()
  vals<-mrEnv$buildevents(raw_data)
  popTable<-mapReduce_reduce( vals,
                              c("riskGroup","sex"),
                              c("sum"),
                              c("n"))
  popTable2<-aggregate(n ~ riskGroup, popTable, sum)
  popTable2$sex<-"All"
  popTable<-rbind(popTable2,popTable)
  popTable3<-aggregate(n ~ sex, popTable, sum)
  names(popTable3)[2]<-"Total"
  popTable<-merge(popTable,popTable3,
                  by = c("sex"),
                  all = TRUE)
  popTable$percentage<-popTable$n*100/popTable$Total
  saveRDS(popTable,"rds/riskTable.RDS")
  popTable<-mapReduce_reduce( vals,
                              c("ldlGroup","sex"),
                              c("sum"),
                              c("n"))
  popTable2<-aggregate(n ~ ldlGroup, popTable, sum)
```

```r
  popTable2$sex<-"All"
  popTable<-rbind(popTable2,popTable)
  popTable3<-aggregate(n ~ sex, popTable, sum)
  names(popTable3)[2]<-"Total"
  popTable<-merge(popTable,popTable3,
                  by = c("sex"),
                  all = TRUE)
  ipopTable<-popTable[popTable$ldlGroup=="3. LDL-C 4 - 5"
                      |popTable$ldlGroup=="4. LDL-C > 5.0",]
  ipopTable<-mapReduce_reduce( ipopTable,
                               c("sex","Total"),
                               c("sum"),
                               c("n"))
  ipopTable$ldlGroup<-"LDL-C > 4.0"
  popTable<-rbind(popTable,ipopTable)
  popTable$percentage<-popTable$n*100/popTable$Total
  saveRDS(popTable,"rds/ldlTable.RDS")

}

if(params$rebuild){
  mrEnv<-new.env()
  NHMStandardLib::mapReduce_setBatchSize(35000)
  source("refSources/supplementalFuncs.R", local=mrEnv)
  raw_data <- mrEnv$getRawData()

  hwparameter_vals<-mrEnv$buildHW1(raw_data)
  hwparameter_vals2<-mrEnv$buildHW2(hwparameter_vals)
  saveRDS(hwparameter_vals2,"rds/hwparameter_vals2.RDS")
  mapReduce_unlinkFileList(hwparameter_vals)
  saveRDS(mrEnv$buildSmoking(raw_data),"rds/smokparameter_vals2.RDS")

  saveRDS(mrEnv$buildSBPHBA1C(raw_data),"rds/hba1csbpTable.RDS")

  saveRDS(mrEnv$builddiab(raw_data),"rds/diabparameter_vals2.RDS")

  saveRDS(mrEnv$buildevents(raw_data),"rds/fatal_fces.RDS")

  saveRDS(mrEnv$buildqalys(raw_data),"rds/eq5dTable.RDS")
  saveRDS(mrEnv$buildhypertenstion_af(raw_data),"rds/hypertenstion_af.RDS")

  mapReduce_unlinkFileList(raw_data)
}
NHMStandardLib::mapReduce_setBatchSize(50000)

if(params$rebuild){
  hwparameter_vals2<-readRDS("rds/hwparameter_vals2.RDS")
  weightTable<-mapReduce_reduce( hwparameter_vals2,
                               c("ageGrp","sex"),
                               c("sum","sum","sum","sum"),
```

```
                               c("HEIGHT","WEIGHT","BMI","n"))
  weightTable$HEIGHT<-weightTable$HEIGHT/weightTable$n
  weightTable$WEIGHT<-weightTable$WEIGHT/weightTable$n
  weightTable$BMI<-weightTable$BMI/weightTable$n
  saveRDS(weightTable,"rds/weightTable.RDS")
  bmiTable<-mapReduce_reduce( hwparameter_vals2,
                               c("riskGroup","age","sex","bmiGrp"),
                               c("sum"),c("n"))

  saveRDS(bmiTable,"rds/bmiTable.RDS")
  rm(list=c("hwparameter_vals2"))

  fatal_fces<-readRDS("rds/fatal_fces.RDS")
  shortenDiag<-function(x){
    retVal<-x
    retVal$cod<-substr(retVal$fcediags, start = 1, stop =
(min(str_length(retVal$fcediags),3)))
    if(inCodeRange(retVal$cod,"I22-I25"))
      retVal$cod<-"I22-I25"
    else if(inCodeRange(retVal$cod,"I60-I64"))
      retVal$cod<-"I60-I63" # because I64 is I60 - I63
    else if(inCodeRange(retVal$cod,"F01-F99"))
      retVal$cod<-"F01-F99"
    return(retVal)
  }
  fatal_fces<-mapReduce_map(fatal_fces,shortenDiag)

  mortTable<-mapReduce_reduce( fatal_fces,
                               c("riskGroup","cod"),
                               c("sum"),c("n"))
  saveRDS(mortTable,"rds/mortTable.RDS")
  dTable<-mapReduce_reduce( fatal_fces,
                          c("riskGroup","age","sex"),
                          c("sum"),c("n"))
  groupPop<-mapReduce_reduce(dTable,
                             c("riskGroup","sex"),
                             c("sum"),c("n"))
  #saveRDS(dTable,"rds/dTable.RDS")
  saveRDS(groupPop,"rds/groupPop.RDS")

  riskgroups<-unique(groupPop$riskGroup)
  gendergroups<-unique(groupPop$sex)
  aliveData<-data.frame()
  for(rg in riskgroups){
    for(z in gendergroups){
      triskGroup=rg
      tsex=z
      taliveData<-data.frame(age=c(0:120),triskGroup,tsex,alive=NA)
      tMortData<-dTable[
```

```
      dTable$riskGroup==triskGroup&dTable$sex==tsex,] %>%
      arrange(age)
    total<-sum(tMortData$n)
    alive<-total
    loc<-1
    for(i in 1:121){
      nextAge<-tMortData[loc,]$age
      taliveData[i,]$alive<-alive
      if(i>nextAge&&alive>0){
        alive<-alive-tMortData[loc,]$n
        loc<-loc+1
      }
    }
    aliveData<-rbind(aliveData,taliveData)
  }
}
aliveData$sex<-NA
aliveData[aliveData$tsex=="Female",]$sex<-"F"
aliveData[aliveData$tsex=="Male",]$sex<-"M"
saveRDS(aliveData,"rds/aliveData.RDS")
setg11nLang("en")

#rm(list=c("fatal_fces"))
tAliveTable<-mapReduce_reduce( aliveData,
                               c("age","tsex"),
                               c("sum"),
                               c("alive"))
names(tAliveTable)<-c("age","sex","alive")
saveRDS(tAliveTable,"rds/tAliveTable.RDS")

smokparameter_vals2<-readRDS("rds/smokparameter_vals2.RDS")

smokeTable<-mapReduce_reduce( smokparameter_vals2,
                              c("age","sex"),
                              c("sum"),
                              c("SMOKING"))
smokeTable<-merge(smokeTable,tAliveTable,
                  by = c("age","sex"),
                  all = TRUE)
saveRDS(smokeTable,"rds/smokeTable.RDS")

diabparameter_vals2<-readRDS("rds/diabparameter_vals2.RDS")
diabCount<-function(x){
  retVal<-x
  retVal$count<-0
  retVal[retVal$DIABETES>0,]$count<-1
  return(retVal)
}
diabparameter_vals3<-mapReduce_map(diabparameter_vals2,diabCount)
```

```r
  rm(list=c("diabparameter_vals2"))
  diabTable<-mapReduce_reduce( diabparameter_vals3,
                              c("age","sex"),
                              c("sum"),
                              c("count"))
  diabTable<-merge(diabTable,tAliveTable,by = c("age","sex"), all = TRUE)
  saveRDS(diabTable,"rds/diabTable.RDS")

  iafTable<-readRDS("rds/hypertenstion_af.RDS")
  iafTable<-mapReduce_reduce( iafTable
                              ,c("age","sex","riskGroup")
                              ,c("sum","sum"),c("I10","I48"))
  names(aliveData)<-c("age","riskGroup","sex","alive","Gender")
  iafTable<-merge(iafTable,aliveData,by = c("age","sex","riskGroup"), all =
TRUE)
  saveRDS(iafTable,"rds/iafTable.RDS")

  hba1csbpTable<-readRDS("rds/hba1csbpTable.RDS")
  hba1csbpTable<-mapReduce_map_toFileList(hba1csbpTable)
  addN<-function(x){
    retVal<-x
    retVal$n<-1
    return(retVal)
  }
  hba1csbpTable<-mapReduce_map_fromtoFileList(hba1csbpTable,addN)
  hba1csbpTable<-mapReduce_reduce_fromFilelist( hba1csbpTable
                              ,c("age","sex","riskGroup")
                              ,c("sum","sum","sum"),c("HBA1C","SBP","n"))
  hba1csbpTable$HBA1C<-hba1csbpTable$HBA1C/hba1csbpTable$n
  hba1csbpTable$SBP<-hba1csbpTable$SBP/hba1csbpTable$n
  hba1csbpTable$HBA1C<-hba1csbpTable$HBA1C/10
  saveRDS(hba1csbpTable,"rds/use_hba1csbpTable.RDS")
}
```

Population

Population at Risk

```
riskTable<-readRDS("rds/riskTable.RDS")
riskTable = riskTable %>% mutate(percentage = paste0(format(round(percentage,
1),decimal.mark="."), "%"))
eT<-pivot_wider(riskTable,id_cols = c(riskGroup),
            names_from = sex,
            values_from = percentage)
names(eT)[1]<-"Risk Group"
eT$`Risk Group`<-str_replace_all(eT$`Risk Group`,"([0-9]{1})(\\.)([0-
9]{1})","\\1.\\3")

eT$`Risk Group`<-str_replace_all(eT$`Risk Group`,">=","≥")
eT$`Risk Group`<-str_replace_all(eT$`Risk Group`," - ","\u2013")
ft <- flextable(eT)
ft <- set_caption(ft,caption="Population at Risk by Risk Group")
ft <- theme_booktabs(ft)
ft<- padding(ft,padding =0,part="all")
#ft <- fontsize(ft, size = 8,part="all")

ft <- autofit(ft)
ft
```

Table S1 presents the distribution of the population across HDL-C/LDL-C risk groups stratified by sex. The largest proportion of the overall population belonged to the low-risk group (HDL-C/LDL-C ≥ 0.45), accounting for 35.9% of individuals, followed by the intermediate and high-risk groups. Females were more frequently represented in lower-risk categories, with 46.6% classified in the low-risk group compared with 25.3% of males. In contrast, males were disproportionately represented in higher-risk categories, particularly in the HDL-C/LDL-C 0.25–0.35 and < 0.25 groups, indicating a less favourable lipid risk profile among men.

Table S1. *Population at Risk by Risk Group.*

Risk Group	All	Female	Male
1. HDL-C/LDL-C ≥ 0.45	35.9%	46.6%	25.3%
2. HDL-C/LDL-C 0.35–0.45	24.9%	25.4%	24.3%
3. HDL-C/LDL-C 0.25–0.35	26.6%	21.3%	32.0%
4. HDL-C/LDL-C < 0.25	12.6%	6.7%	18.5%

```
ldlTable<-readRDS("rds/ldlTable.RDS")
ldlTable = ldlTable %>% mutate(percentage = paste0(format(round(percentage,
1),decimal.mark="."), "%"))
eT<-pivot_wider(ldlTable,id_cols = c(ldlGroup),
            names_from = sex,
            values_from = percentage)
names(eT)[1]<-"Risk Group"
eT$`Risk Group`<-str_replace_all(eT$`Risk Group`,"([0-9]{1})(\\.)([0-
9]{1})","\\1.\\3")
```

```r
eT$`Risk Group`<-str_replace_all(eT$`Risk Group`,">=","≥")
eT$`Risk Group`<-str_replace_all(eT$`Risk Group`," - ","\u2013")
eT<-eT[c(1,2,5,3,4),]
ft <- flextable(eT)
ft <- set_caption(ft,caption="Population at Risk by LDL Group")
ft <- theme_booktabs(ft)
ft<- padding(ft,padding =0,part="all")
#ft <- fontsize(ft, size = 8,part="all")

ft <- autofit(ft)
ft
```

Table S2 presents the distribution of the population according to LDL-C categories stratified by sex. The population was relatively evenly distributed across LDL-C categories, with approximately one-third of individuals classified in the LDL-C < 3.1 mmol/L (low risk) and LDL-C 3.1–4.0 mmol/L groups. Females were more frequently represented in lower LDL-C categories, whereas males showed a higher proportion in elevated LDL-C categories, particularly LDL-C > 4.0 mmol/L and LDL-C 4–5 mmol/L. The proportion of individuals with very high LDL-C levels > 5.0 mmol/L (highest risk) was low in both sexes, accounting for approximately 2.5% of the population.

***Table S2.** Population at Risk by LDL Group.*

Risk Group	All	Female	Male
1. LDL-C < 3.1	34.1%	36.5%	31.8%
2. LDL-C 3.1–4	34.4%	34.9%	33.8%
LDL-C > 4.0	31.5%	28.6%	34.4%
3. LDL-C 4–5	29.0%	26.3%	31.7%
4. LDL-C > 5.0	2.5%	2.3%	2.7%

Heights and Weights

```r
weightTable<-readRDS("rds/weightTable.RDS")
bmiTable<-readRDS("rds/bmiTable.RDS")

eT<-pivot_wider(weightTable,id_cols = c(ageGrp),
                names_from = sex,
                values_from = c("HEIGHT","WEIGHT","BMI"))
teT<-eT %>% mutate_if(is.numeric, round,digits=2)
teT<-teT %>% mutate_if(is.numeric, format,big.mark=",",decimal.mark=".")
names(teT)[1]="Age Group"
ft <- flextable(teT)
ft <- set_caption(ft,caption="Population heights and weights")
ft <- separate_header(ft)
ft <- autofit(ft)
ft
```

Table S3 presents height, weight and BMI per gender and age group.

***Table S3.** Population heights and weights.*

Age Group	HEIGHT		WEIGHT		BMI	
	Female	Male	Female	Male	Female	Male
00 - 04	83.42	88.19	15.29	00 - 04	83.42	88.19
05 - 09	117.65	120.47	33.26	05 - 09	117.65	120.47
10 - 14	150.29	150.43	51.23	10 - 14	150.29	150.43
15 - 19	163.36	173.39	67.34	15 - 19	163.36	173.39
20 - 24	163.35	176.13	71.05	20 - 24	163.35	176.13
25 - 29	163.36	176.14	71.84	82.21	27.07	26.62
30 - 34	163.36	176.14	72.34	83.93	27.22	27.15
35 - 39	163.37	176.15	72.71	85.25	27.32	27.55
40 - 44	163.37	176.16	72.97	86.28	27.39	27.86
45 - 49	162.74	175.53	73.13	87.09	27.64	28.30
50 - 54	161.68	174.47	72.93	87.34	27.92	28.72
55 - 59	160.62	173.42	71.43	85.72	27.69	28.52
60 - 64	159.57	172.36	68.81	82.78	27.01	27.87
65 - 69	158.52	171.32	66.27	79.96	26.34	27.23
70 - 74	157.48	170.28	64.04	77.42	25.78	26.68
75 - 79	156.44	169.25	62.12	75.18	25.33	26.21
80 and older	154.56	167.47	59.40	71.79	24.79	25.54

BMI by age and sex

```
rgroups<-unique(bmiTable$riskGroup)
bmiTable2<-bmiTable[bmiTable$riskGroup==rgroups[[1]]&bmiTable$sex=="Female",]
bmiTable2<-bmiTable2[bmiTable2$age>=20,]
bmiTable2$Legend<-bmiTable2$bmiGrp
multilineAreaPlot(bmiTable2,"","Age","Population (n)",age,n,textSize=12)
```

Figure S1 presents the age-specific distribution of the female population by BMI category among individuals with an HDL-C/LDL-C ratio ≥ 0.45 (low risk category). Overweight and obese categories account for the largest proportion of the population across most age groups, while the underweight category contributes minimally. Overall, the figure demonstrates age-related changes in both population size and BMI distribution.

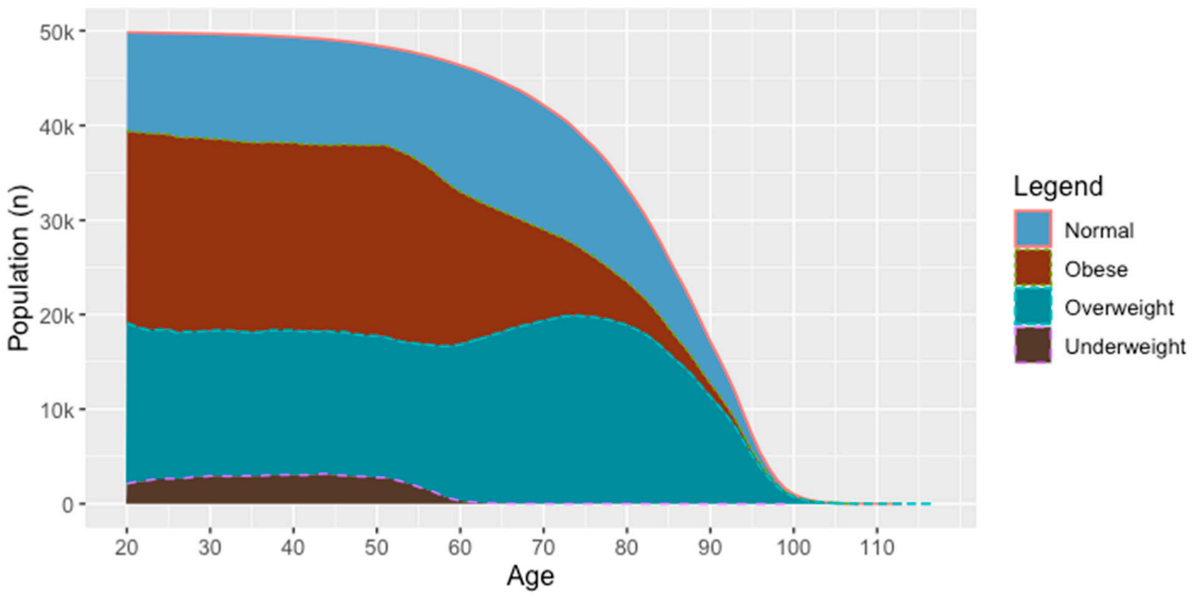

Figure S1. *Female BMI, HDL-C/LDL-C ≥ 0.45.*

```
rgroups<-unique(bmiTable$riskGroup)
bmiTable2<-bmiTable[bmiTable$riskGroup==rgroups[[2]]&bmiTable$sex=="Female",]
bmiTable2<-bmiTable2[bmiTable2$age>=20,]
bmiTable2$Legend<-bmiTable2$bmiGrp
multilineAreaPlot(bmiTable2,"","Age","Population (n)",age,n,textSize=12)
```

The Figure S2 presents the age-specific distribution of the female population by BMI category among individuals with an HDL-C/LDL-C ratio of 0.35–0.45 (moderate risk category). Overweight and obese categories account for the largest proportion of the population across most age groups, while the underweight category remains relatively small. Obesity becomes less prevalent in older age groups, while overweight population increases with age.

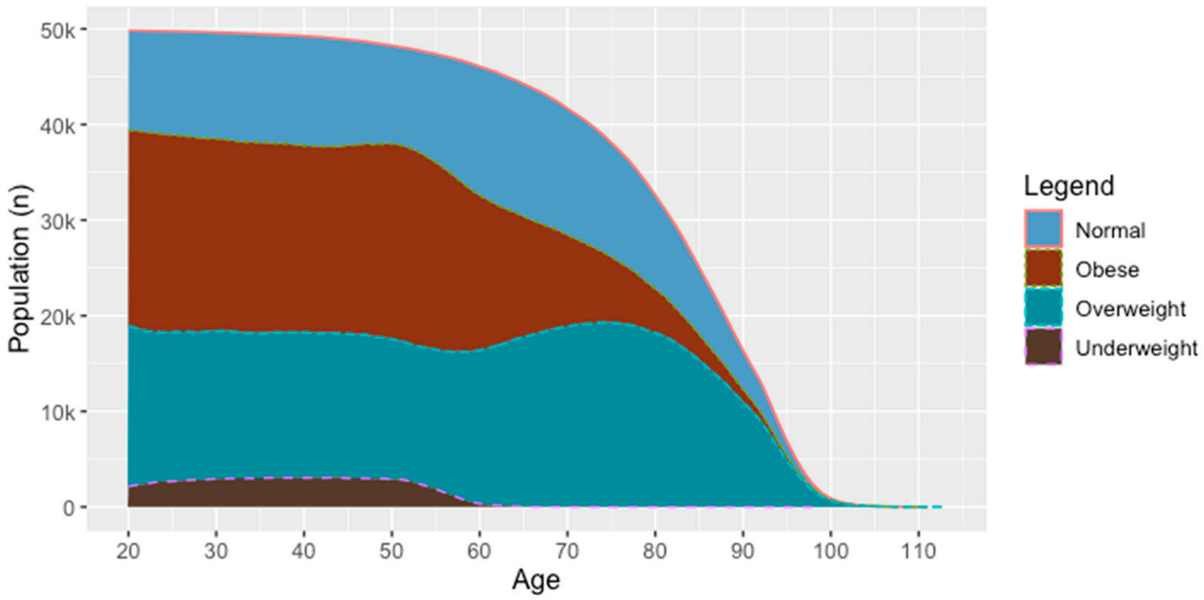

Figure S2. *Female BMI, HDL-C/LDL-C 0.35 – 0.45.*

```r
rgroups<-unique(bmiTable$riskGroup)
bmiTable2<-bmiTable[bmiTable$riskGroup==rgroups[[3]]&bmiTable$sex=="Female",]
bmiTable2<-bmiTable2[bmiTable2$age>=20,]
bmiTable2$Legend<-bmiTable2$bmiGrp
multilineAreaPlot(bmiTable2,"","Age","Population (n)",age,n,textSize=12)
```

The figure S3 illustrates the age-specific distribution of the female population across BMI categories among individuals with an HDL-C/LDL-C ratio < 0.35 (high risk and very high risk categories). Overweight and obese groups constitute the largest share of the population throughout most age ranges, while the underweight category remains minimal. In older age groups, the relative contribution of the overweight category becomes more pronounced, whereas obesity shows a progressive decline with advancing age.

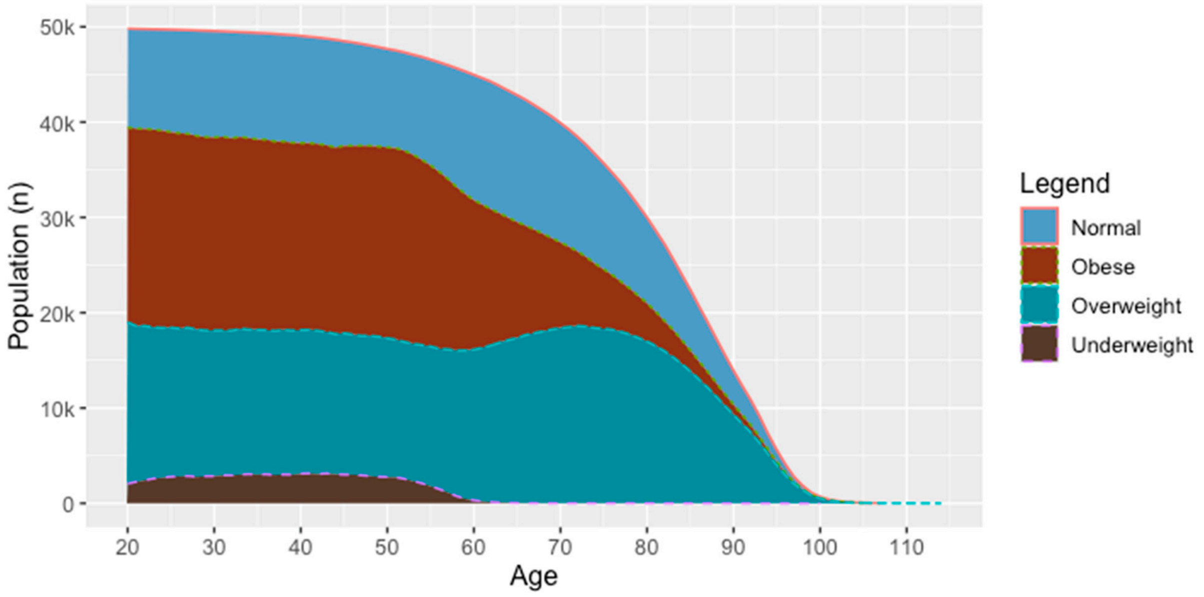

Figure S3. *Female BMI, HDL-C/LDL-C < 0.35.*

```
rgroups<-unique(bmiTable$riskGroup)
bmiTable2<-bmiTable[bmiTable$riskGroup==rgroups[[1]]&bmiTable$sex=="Male",]
bmiTable2<-bmiTable2[bmiTable2$age>=20,]
bmiTable2$Legend<-bmiTable2$bmiGrp
multilineAreaPlot(bmiTable2,"","Age","Population (n)",age,n,textSize=12)
```

The figure S4 illustrates the age-specific distribution of the male population across BMI categories among individuals with an HDL-C/LDL-C ratio ≥ 0.45 (low risk category). Overweight and obese groups account for the largest proportion of the population across most age groups, while the underweight category remains negligible. Obesity is more prominent in younger and middle-aged individuals, whereas the relative contribution of the overweight category becomes more pronounced in older age groups.

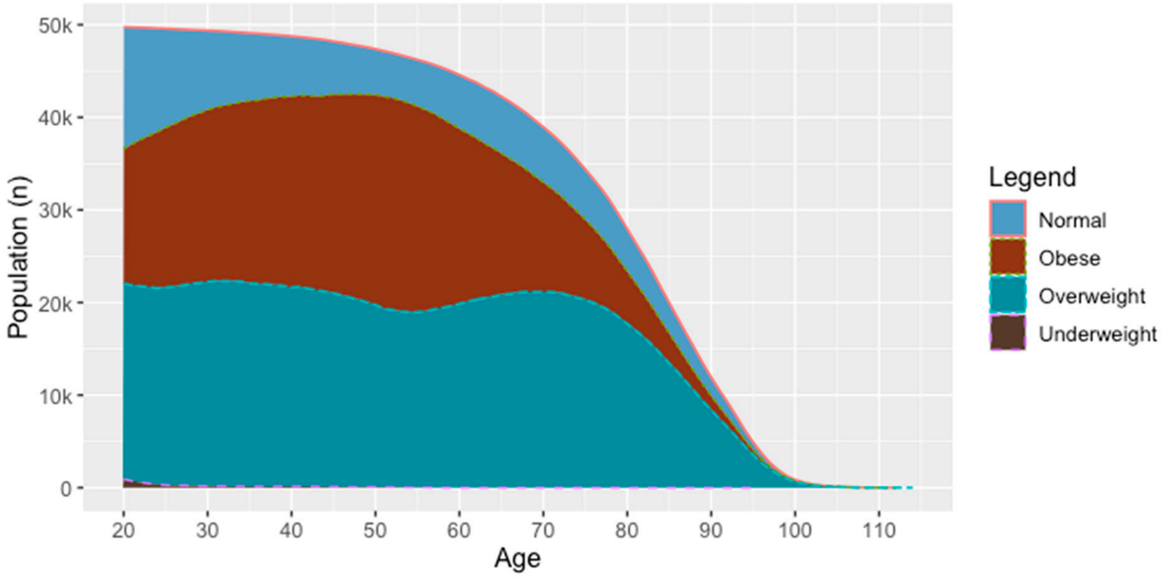

Figure S4. *Male BMI, HDL-C/LDL-C ≥ 0.45.*

```r
rgroups<-unique(bmiTable$riskGroup)
bmiTable2<-bmiTable[bmiTable$riskGroup==rgroups[[2]]&bmiTable$sex=="Male",]
bmiTable2<-bmiTable2[bmiTable2$age>=20,]
bmiTable2$Legend<-bmiTable2$bmiGrp
multilineAreaPlot(bmiTable2,"","Age","Population (n)",age,n,textSize=12)
```

The figure S5 illustrates the age-specific distribution of the male population across BMI categories among individuals with an HDL-C/LDL-C ratio of 0.35–0.45 (moderate risk category). Obese and overweight categories dominate across nearly all age groups, together accounting for the majority of the population throughout adulthood. The normal BMI category contributes a smaller but consistent proportion across the lifespan, while the underweight category is negligible.

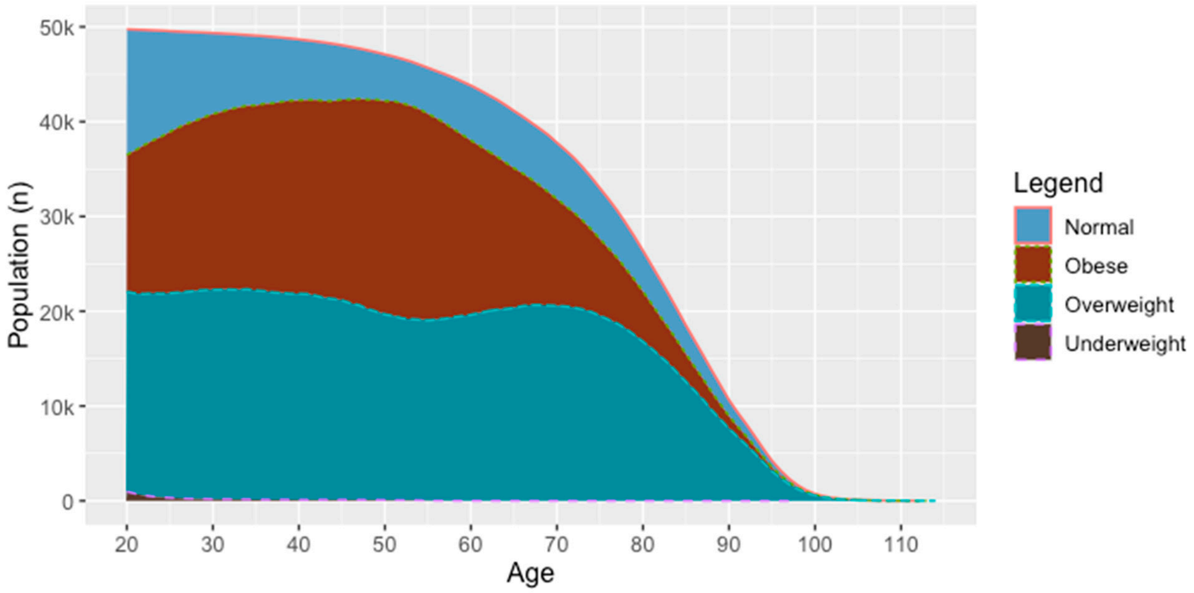

Figure S5. *Male BMI, HDL-C/LDL-C 0.35–0.45.*

```r
rgroups<-unique(bmiTable$riskGroup)
bmiTable2<-bmiTable[bmiTable$riskGroup==rgroups[[3]]&bmiTable$sex=="Male",]
bmiTable2<-bmiTable2[bmiTable2$age>=20,]
bmiTable2$Legend<-bmiTable2$bmiGrp
multilineAreaPlot(bmiTable2,"","Age","Population (n)",age,n,textSize=12)
```

The Figure S6 illustrates the age-specific distribution of the male population across BMI categories among individuals with an HDL-C/LDL-C ratio < 0.35 (high risk and very high risk categories). Obese and overweight categories dominate throughout most age groups, together accounting for the majority of the population across adulthood. The normal BMI category represents a smaller proportion, while the underweight category remains minimal across all ages.

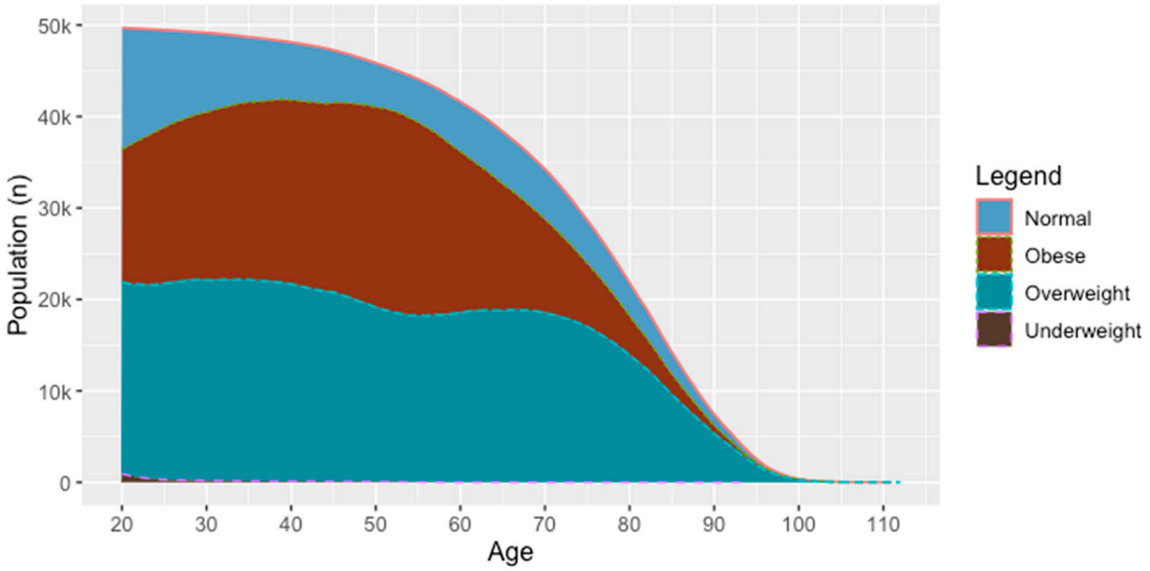

Figure S6. *Male BMI, HDL-C/LDL-C < 0.35.*

Population Pyramids

```
#dTable<-readRDS("rds/dTable.RDS")
groupPop<-readRDS("rds/groupPop.RDS")
riskgroups<-unique(groupPop$riskGroup)
aliveData<-readRDS("rds/aliveData.RDS")

data <- data.frame(
  age=aliveData[aliveData$triskGroup==riskgroups[[1]],]$age,
  sex=aliveData[aliveData$triskGroup==riskgroups[[1]],]$sex,
  Population=aliveData[aliveData$triskGroup==riskgroups[[1]],]$alive)
populationPyramid(data,CONCAT("Population Risk Group
",str_replace_all(riskgroups[[1]],"([0-9]{1})(\\.)([0-9]{1})","\\1.\\3")))
```

The Figure S7 presents the population pyramid for the low-risk group defined by an HDL-C/LDL-C ratio ≥ 0.45 (low risk category), stratified by age and sex. The population size is highest among younger and middle-aged individuals and progressively decreases with advancing age. Male and female population distributions appear broadly symmetrical across most age groups, with only minor sex-related differences. A pronounced decline in population size is observed after approximately 75–80 years of age.

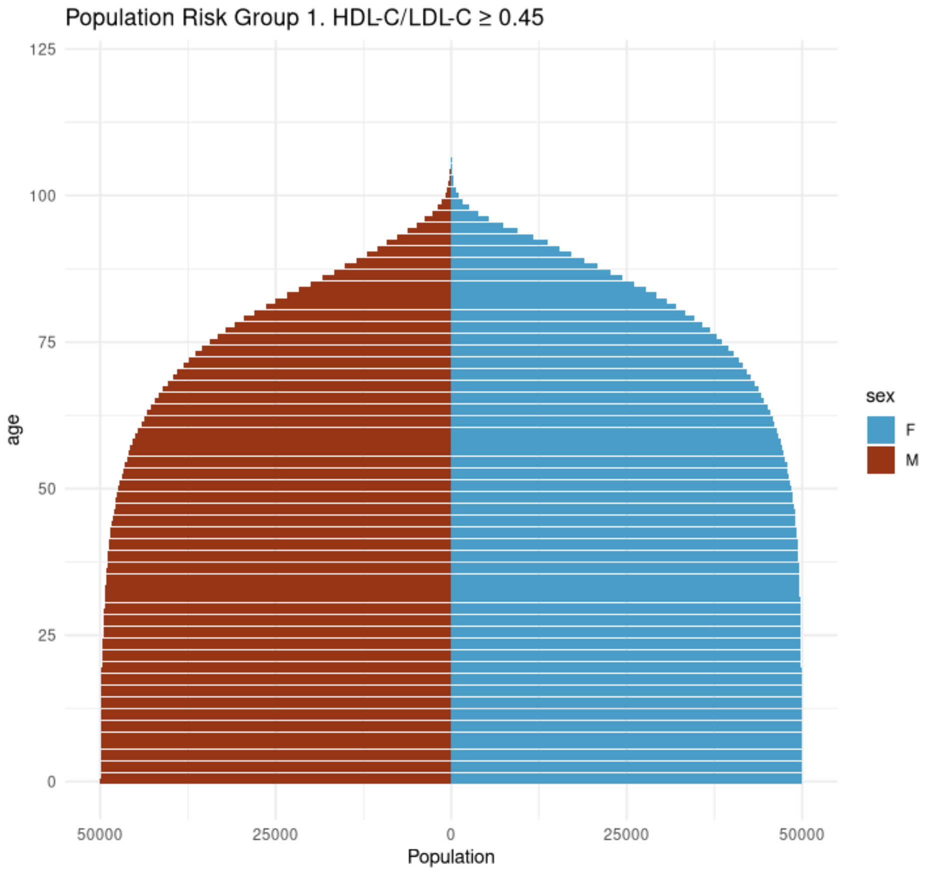

Figure S7. *Low Risk Population pyramid.*

```
data <- data.frame(
  age=aliveData[aliveData$triskGroup==riskgroups[[2]],]$age,
  sex=aliveData[aliveData$triskGroup==riskgroups[[2]],]$sex,
  Population=aliveData[aliveData$triskGroup==riskgroups[[2]],]$alive)
populationPyramid(data,CONCAT("Population Risk Group
",str_replace_all(riskgroups[[2]],"([0-9]{1})(\\.)([0-9]{1})","\\1.\\3")))
```

The Figure S8 presents the population pyramid for the intermediate-risk group defined by an HDL-C/LDL-C ratio of 0.35–0.45 (moderate risk category), stratified by age and sex. The population is largest among younger and middle-aged individuals and gradually declines with increasing age. Male and female population distributions are broadly symmetrical across most age groups, with only minor differences between sexes.

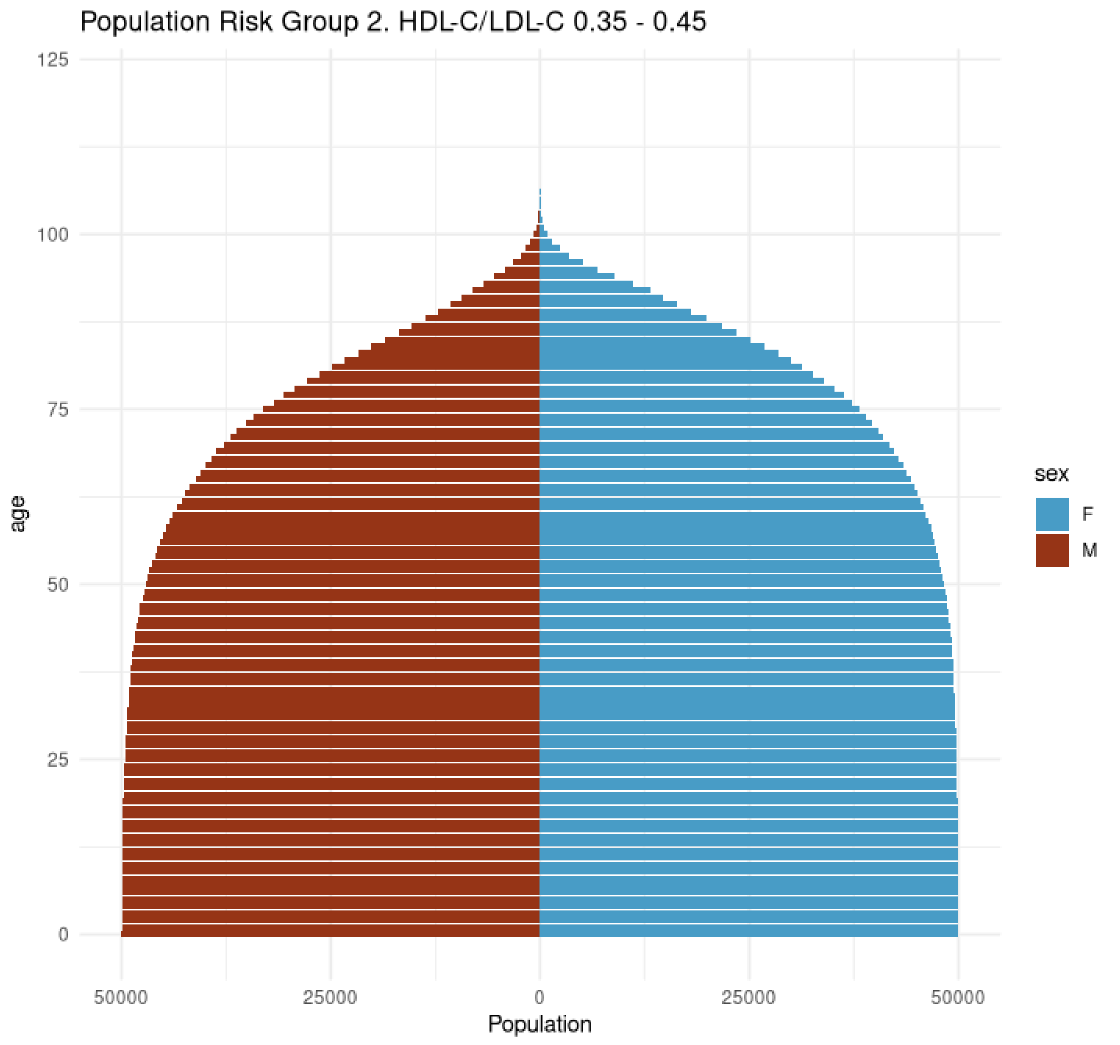

Figure S8. *Moderate risk population pyramid.*

```
data <- data.frame(
  age=aliveData[aliveData$triskGroup==riskgroups[[3]],]$age,
  sex=aliveData[aliveData$triskGroup==riskgroups[[3]],]$sex,
  Population=aliveData[aliveData$triskGroup==riskgroups[[3]],]$alive)
```

```
populationPyramid(data,CONCAT("Population Risk Group
",str_replace_all(riskgroups[[3]],"([0-9]{1})(\\.)([0-9]{1})","\\1.\\3")))
```

The Figure S9 presents the population pyramid for the high-risk group defined by an HDL-C/LDL-C ratio of 0.25–0.35 (high risk category), stratified by age and sex. The population is concentrated in younger and middle-aged groups, with a gradual decline observed with advancing age. Male and female distributions are broadly symmetrical across the age spectrum, with only minor differences between sexes after age 50.

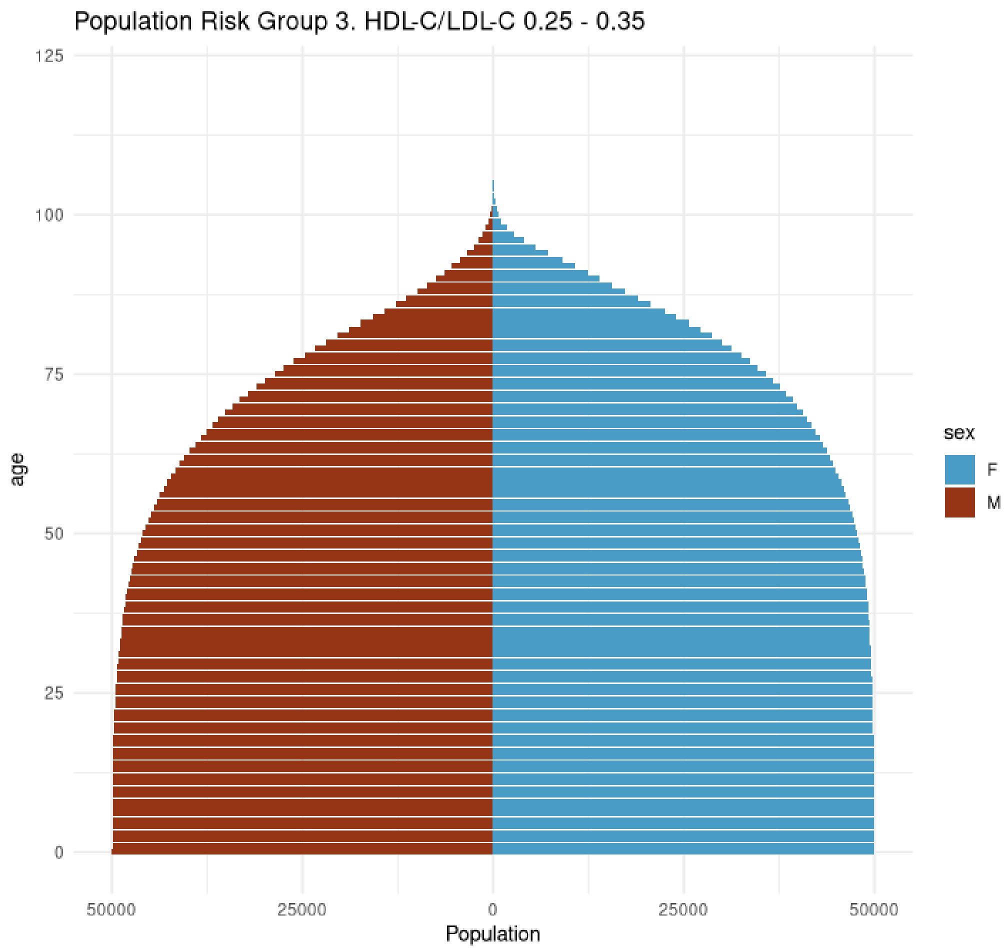

Figure S9. *High Risk population pyramid.*

```r
data <- data.frame(
  age=aliveData[aliveData$triskGroup==riskgroups[[4]],]$age,
  sex=aliveData[aliveData$triskGroup==riskgroups[[4]],]$sex,
  Population=aliveData[aliveData$triskGroup==riskgroups[[4]],]$alive)
populationPyramid(data,CONCAT("Population Risk Group
",str_replace_all(riskgroups[[4]],"([0-9]{1})(\\.)([0-9]{1})","\\1.\\3")))
```

Figure S10 presents the population pyramid for the very-high-risk group defined by an HDL-C/LDL-C ratio < 0.25, stratified by age and sex. The population is predominantly concentrated in younger and middle-aged individuals, with a progressive decline in population size at older ages. Male and female distributions remain broadly symmetrical until approximately 40 years of age, after which the overall pyramid becomes noticeably narrower in older age groups compared with lower-risk populations. A marked reduction in population size is observed after approximately 65–75 years of age, likely reflecting earlier cardiovascular mortality associated with elevated cumulative cardiometabolic burden.

This pattern may also help explain the paradoxical findings observed for lifetime ischaemic stroke burden within the very-high-risk population. Although cardiovascular risk is substantially elevated in this group, earlier mortality from myocardial infarction and other cardiovascular diseases reduces the probability of surviving into older ages where stroke incidence becomes more prevalent, illustrating the complex relationship between lipid imbalance, survival trajectories, and cumulative lifetime cardiovascular outcomes.

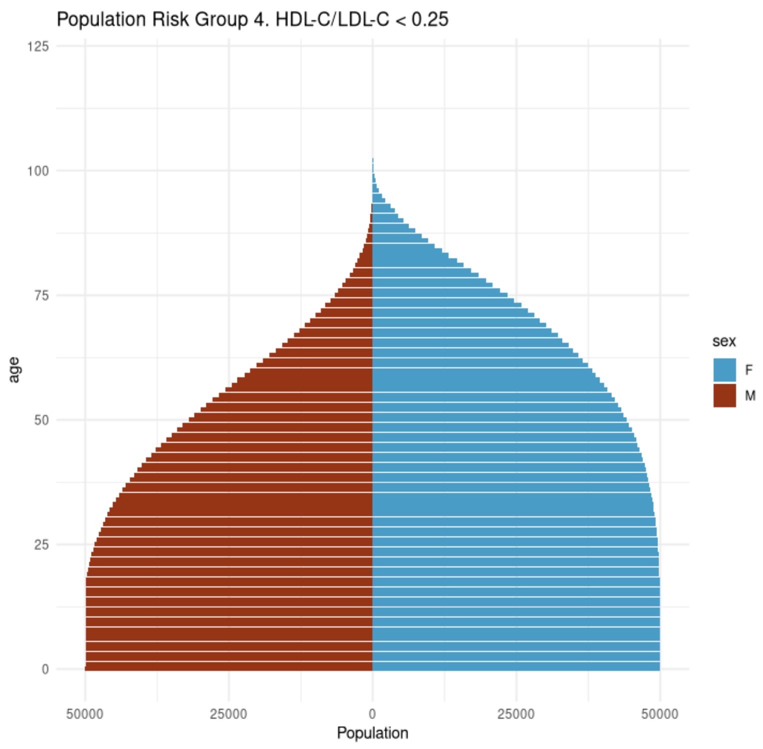

Figure S10. Very High Risk population pyramid.

Risk Factors

Smoking

```
smokeTable<-readRDS("rds/smokeTable.RDS")
smokeTable[is.na(smokeTable$SMOKING),]<-0
smokeTable<-smokeTable[smokeTable$age<=95,]
smokeTable<-smokeTable[smokeTable$SMOKING>100,]
smokeTable$prevalence<-smokeTable$SMOKING*100/smokeTable$alive
smokeTable$Legend<-smokeTable$sex
multilinePlot(smokeTable,"","Age","Prevalence
(%)",age,prevalence,textSize=12)
```

The Figure S11 depicts age- and sex-specific smoking prevalence across the lifespan. Smoking prevalence increases rapidly from adolescence into early adulthood in both sexes, reaching a peak at approximately 25 years of age. Peak prevalence is substantially higher among males, approaching 22–23%, compared with approximately 8–9% among females. After early adulthood, smoking prevalence gradually declines with increasing age in both sexes; however, males consistently maintain higher prevalence rates throughout all age groups. By older age, smoking prevalence decreases to approximately 7–8% among males and 3% among females.

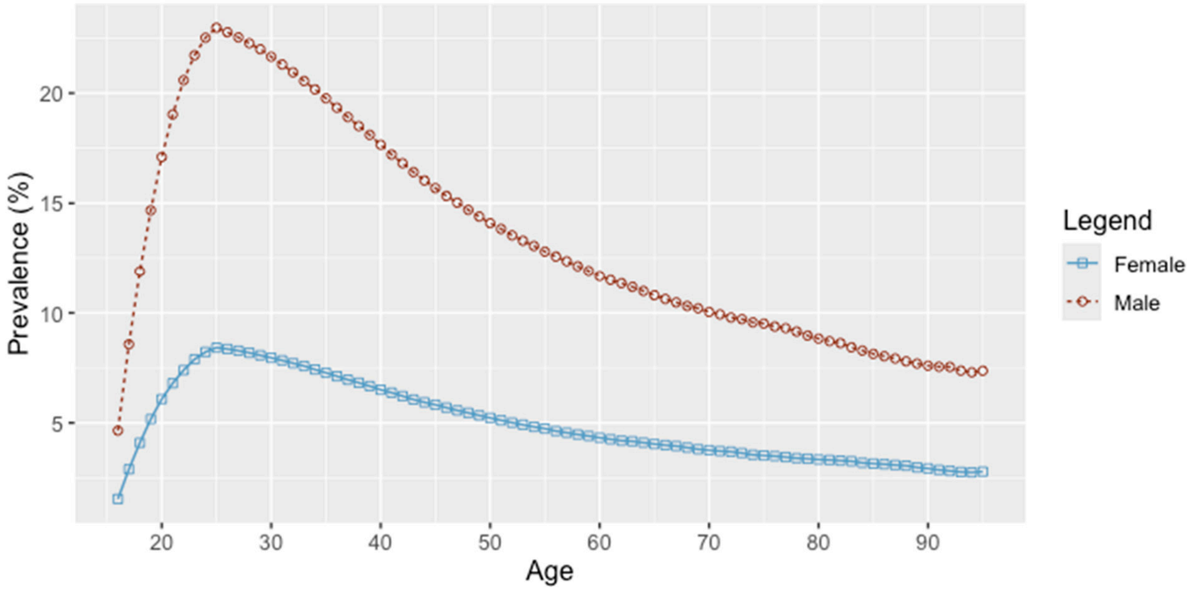

Figure S11. *Smoking Prevalence.*

Diabetes

```
diabTable<-readRDS("rds/diabTable.RDS")
diabTable[is.na(diabTable$count),]<-0
diabTable<-diabTable[diabTable$age<=95,]
diabTable<-diabTable[diabTable$count>100,]
diabTable$prevalence<-diabTable$count*100/diabTable$alive
diabTable$Legend<-diabTable$sex
multilinePlot(diabTable,"","Age","Prevalence (%)",age,prevalence,textSize=12)
```

The Figure S12 depicts age- and sex-specific diabetes prevalence across the lifespan. Diabetes prevalence increases progressively with age in both sexes, rising from below 1% in younger individuals to approximately 20% by 65–75 years of age. Prevalence is slightly higher among females than males across most age groups, although differences between sexes remain relatively small. After reaching peak levels in older adulthood, diabetes prevalence gradually declines in the oldest age groups, decreasing to approximately 15% by 90 years of age.

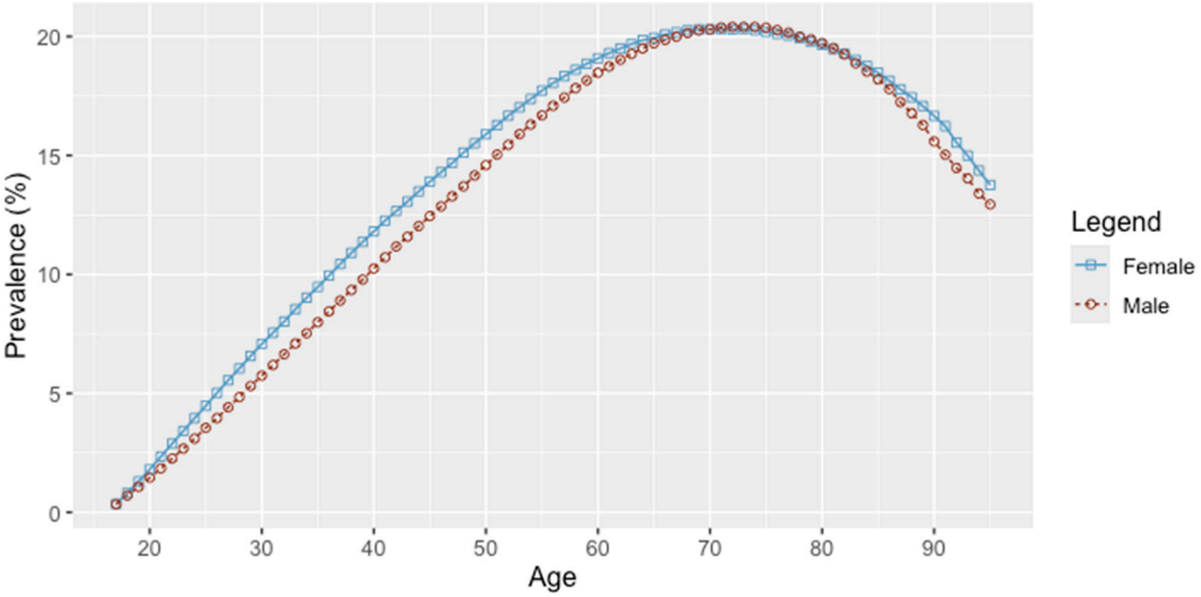

Figure S12. *Diabetes Prevalence.*

HbA1c

```
hba1csbpTable<-readRDS("rds/use_hba1csbpTable.RDS")
hba1csbpTablev<-hba1csbpTable[hba1csbpTable$age<=95,]
hba1csbpTablev<-hba1csbpTablev[hba1csbpTablev$age>=18,]
hba1csbpTablev<-hba1csbpTablev[hba1csbpTablev$sex=="Female",]
hba1csbpTablev$Legend<-hba1csbpTablev$riskGroup
multilinePlot(hba1csbpTablev,"","Age","Mean HbA1c",age,HBA1C,textSize=12)
```

The Figure S13 presents mean HbA1c levels across age groups in females stratified by HDL-C/LDL-C risk group. Mean HbA1c increases progressively from early adulthood, reaching peak levels around 65–75 years of age, followed by a gradual decline in older age groups. Females in lower-risk groups (HDL-C/LDL-C ≥ 0.35) consistently demonstrate higher mean HbA1c values compared with higher-risk groups, while the very high-risk group (HDL-C/LDL-C < 0.25) shows the lowest HbA1c levels across all ages. HbA1c in the very high risk group declines rapidly due to high mortality rates in people with both high HbA1c and HDL-C/LDL-C risk. Differences between risk groups become more pronounced with advancing age, particularly after approximately 50 years of age.

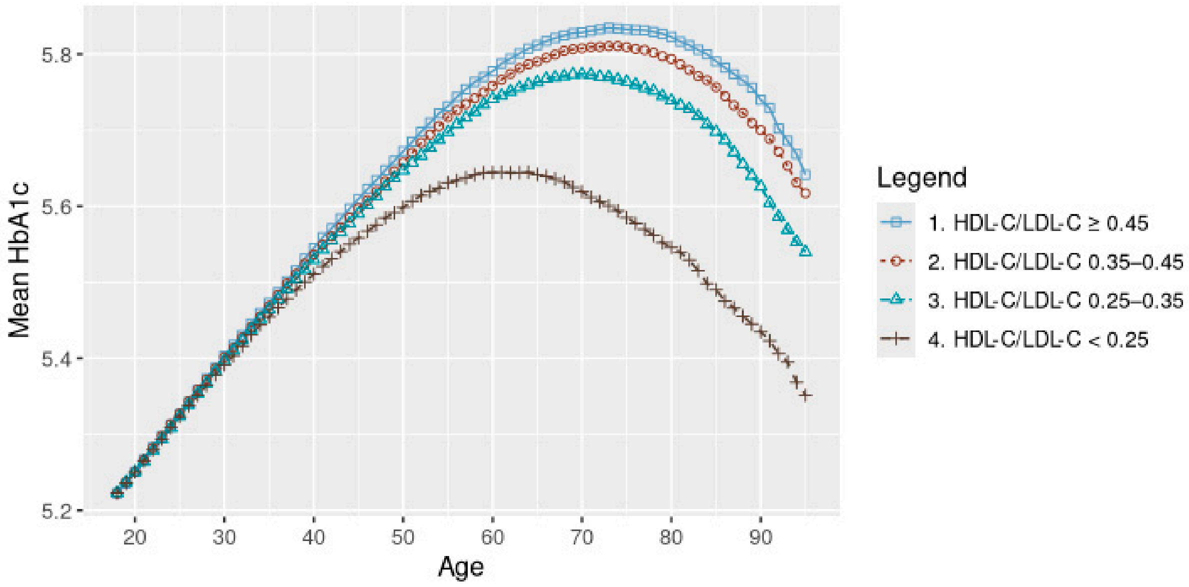

Figure S13. *Female HbA1c by Risk Group.*

```
hba1csbpTablev<-hba1csbpTable[hba1csbpTable$sex=="Male",]
hba1csbpTablev<-hba1csbpTablev[hba1csbpTablev$age<=95,]
hba1csbpTablev<-hba1csbpTablev[hba1csbpTablev$age>=18,]
hba1csbpTablev$Legend<-hba1csbpTablev$riskGroup
```

```r
hba1csbpTablev$Legend<-gsub(">=","≥",hba1csbpTablev$Legend)
hba1csbpTablev$Legend<-gsub(" - ","\u2013",hba1csbpTablev$Legend)
multilinePlot(hba1csbpTablev,"","Age","Mean HbA1c",age,HBA1C,textSize=12)
```

The Figure S14 presents mean HbA1c levels across age groups in males stratified by HDL-C/LDL-C risk group. Mean HbA1c increases steadily from early adulthood, reaching peak values around 70–75 years of age, followed by a gradual decline in older age groups.

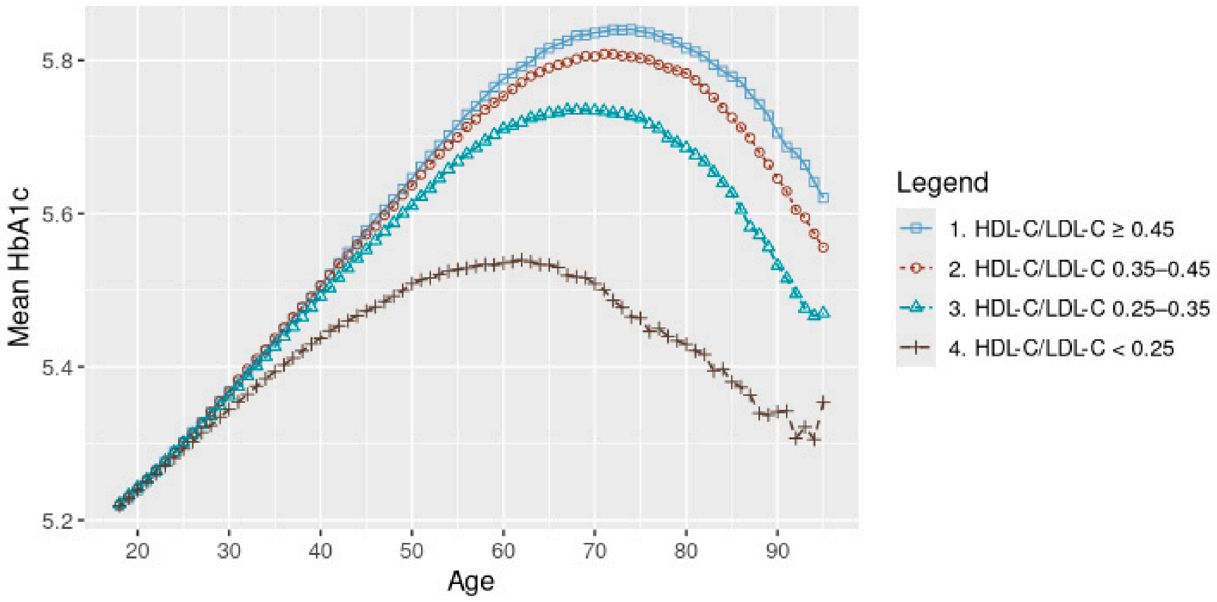

Figure S14. *Male HbA1c by Risk Group.*

Hypertension

```
iafTable<-readRDS("rds/iafTable.RDS")
iafTablev<-iafTable[iafTable$age<=95,]
iafTablev<-iafTablev[iafTablev$sex=="Female",]
iafTablev$Legend<-iafTablev$riskGroup

iafTablev$Legend<-gsub(">=","≥",iafTablev$Legend)
iafTablev$Legend<-gsub(" - ","\u2013",iafTablev$Legend)
iafTablev$prevalence<-iafTablev$I10*100/iafTablev$alive
multilinePlot(iafTablev,"","Age","Prevalence (%)",age,prevalence,textSize=12)
```

The Figure S15 presents age-specific hypertension prevalence in females stratified by HDL-C/LDL-C risk group. Hypertension prevalence remains very low in early life and begins to increase markedly after approximately 30–40 years of age. Prevalence rises progressively with advancing age, reaching approximately 60% in the oldest age groups. Differences between risk groups are relatively small across most ages, although females in the very high-risk group (HDL-C/LDL-C < 0.25) tend to show slightly lower hypertension prevalence in older age groups compared with the other risk categories.

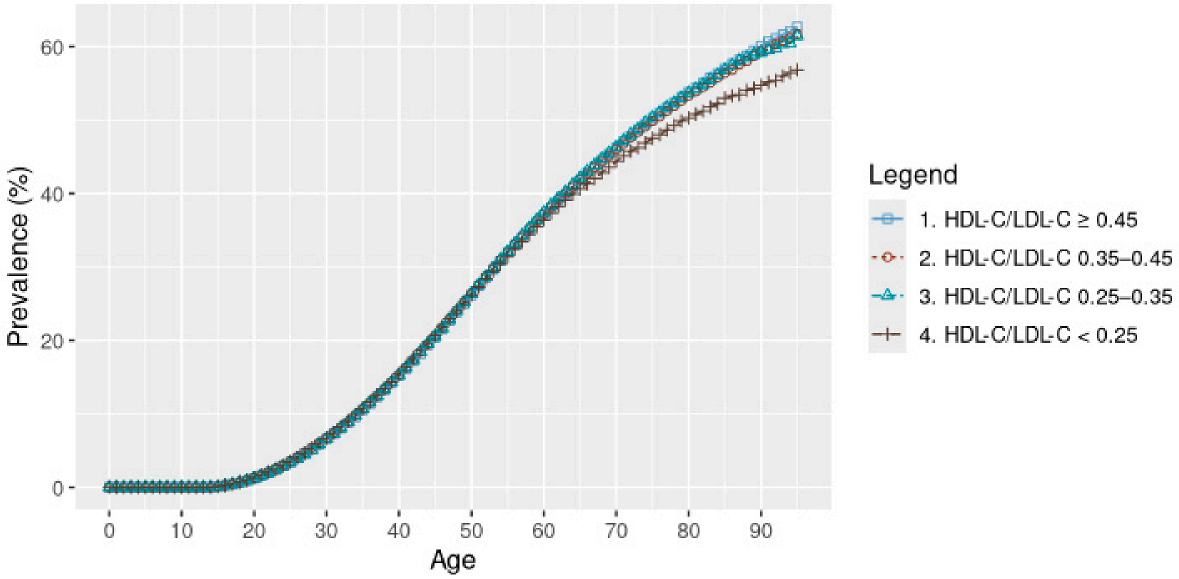

Figure S15. *Female Hypertension Prevalence by Risk Group.*

```
iafTablev<-iafTable[iafTable$age<=95,]
iafTablev<-iafTablev[iafTablev$sex=="Male",]
iafTablev$Legend<-iafTablev$riskGroup

iafTablev$Legend<-gsub(">=","≥",iafTablev$Legend)
iafTablev$Legend<-gsub(" - ","\u2013",iafTablev$Legend)
```

```
iafTablev$prevalence<-iafTablev$I10*100/iafTablev$alive
multilinePlot(iafTablev,"","Age","Prevalence (%)",age,prevalence,textSize=12)
```

The Figure S16 presents age-specific hypertension prevalence in males stratified by HDL-C/LDL-C risk group. Hypertension prevalence remains minimal during early life and increases substantially after approximately 30–40 years of age. Prevalence rises progressively with advancing age, reaching approximately 60% in older age groups among most risk categories. Males in the very high-risk group (HDL-C/LDL-C < 0.25) demonstrate consistently lower hypertension prevalence compared with the other groups.

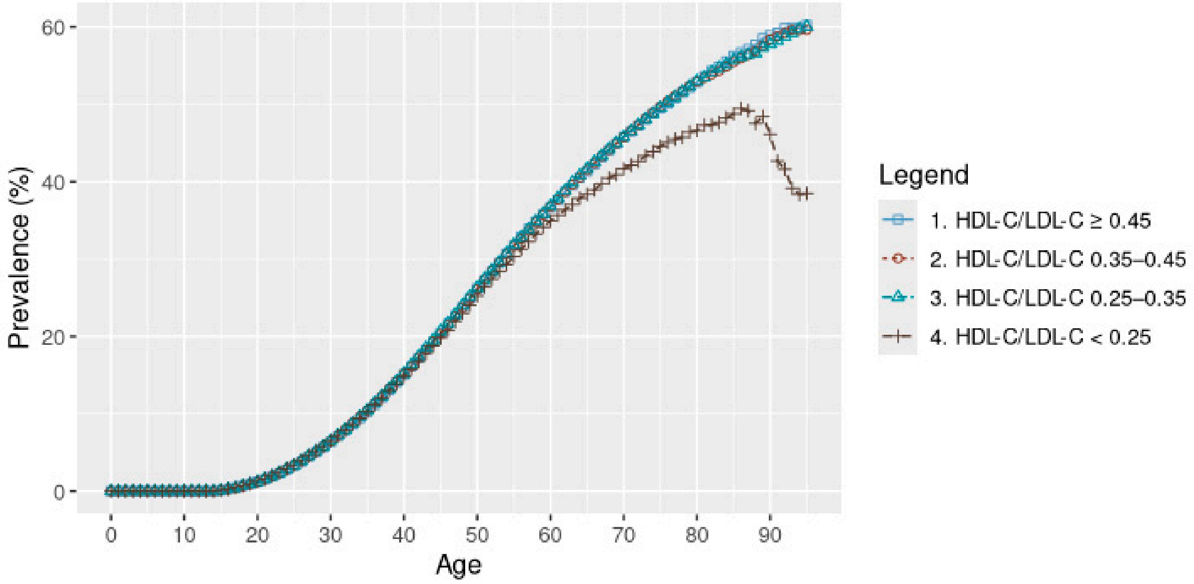

Figure S16. *Male Hypertension Prevalence by Risk Group.*

Atrial Fibralation

```
iafTablev<-iafTable[iafTable$age<=95,]
iafTablev<-iafTablev[iafTablev$sex=="Female",]
iafTablev$Legend<-iafTablev$riskGroup

iafTablev$Legend<-gsub(">=","≥",iafTablev$Legend)
iafTablev$Legend<-gsub(" - ","\u2013",iafTablev$Legend)
iafTablev$prevalence<-iafTablev$I48*100/iafTablev$alive
multilinePlot(iafTablev,"","Age","Prevalence (%)",age,prevalence,textSize=12)
```

The Figure S17 presents age-specific atrial fibrillation prevalence in females stratified by HDL-C/LDL-C risk group. Prevalence remains close to zero throughout early and middle adulthood and begins to increase gradually after approximately 40–50 years of age. A marked rise is observed after 70 years of age, with prevalence exceeding 10% in the oldest age groups. Differences between risk groups are relatively small at younger ages but become more noticeable in older individuals, with the very high-risk group (HDL-C/LDL-C < 0.25) demonstrating the highest prevalence in advanced age.

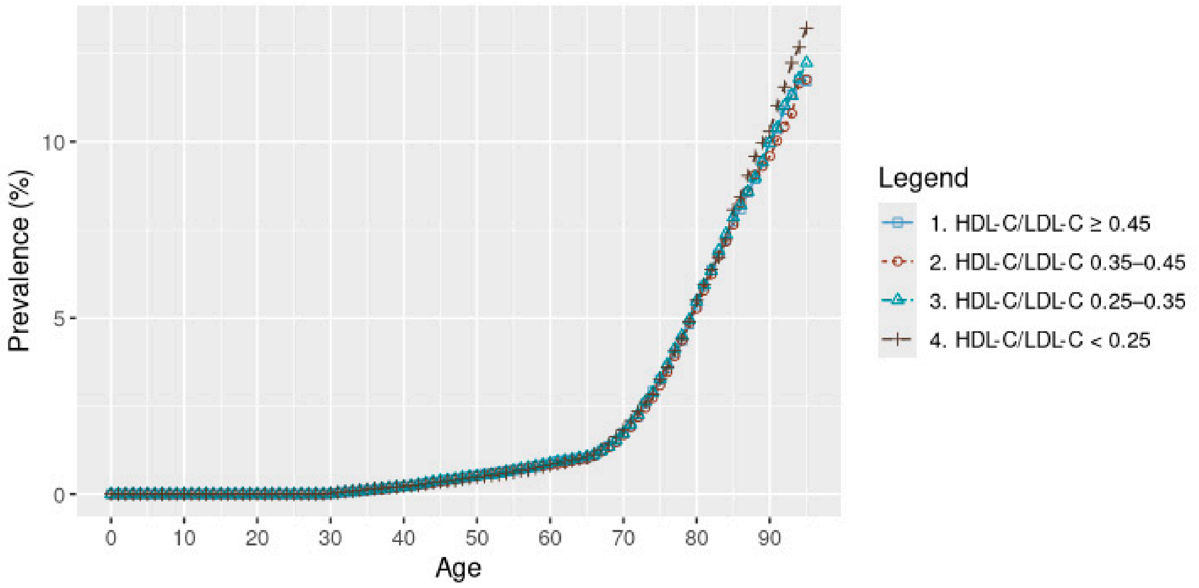

Figure S17. *Female Atrial Fibrilation Prevalence by Risk Group.*

```
iafTablev<-iafTable[iafTable$age<=95,]
iafTablev<-iafTablev[iafTablev$sex=="Male",]
iafTablev$Legend<-iafTablev$riskGroup

iafTablev$Legend<-gsub(">=","≥",iafTablev$Legend)
iafTablev$Legend<-gsub(" - ","\u2013",iafTablev$Legend)

iafTablev$prevalence<-iafTablev$I48*100/iafTablev$alive
multilinePlot(iafTablev,"","Age","Prevalence (%)",age,prevalence,textSize=12)
```

The Figure S18 presents age-specific atrial fibrillation prevalence in males stratified by HDL-C/LDL-C risk group. Prevalence remains very low during early and middle adulthood, followed by a gradual increase after approximately 40–50 years of age. A pronounced rise is observed after 70 years of age, with prevalence reaching approximately 10–14% in the oldest age groups.

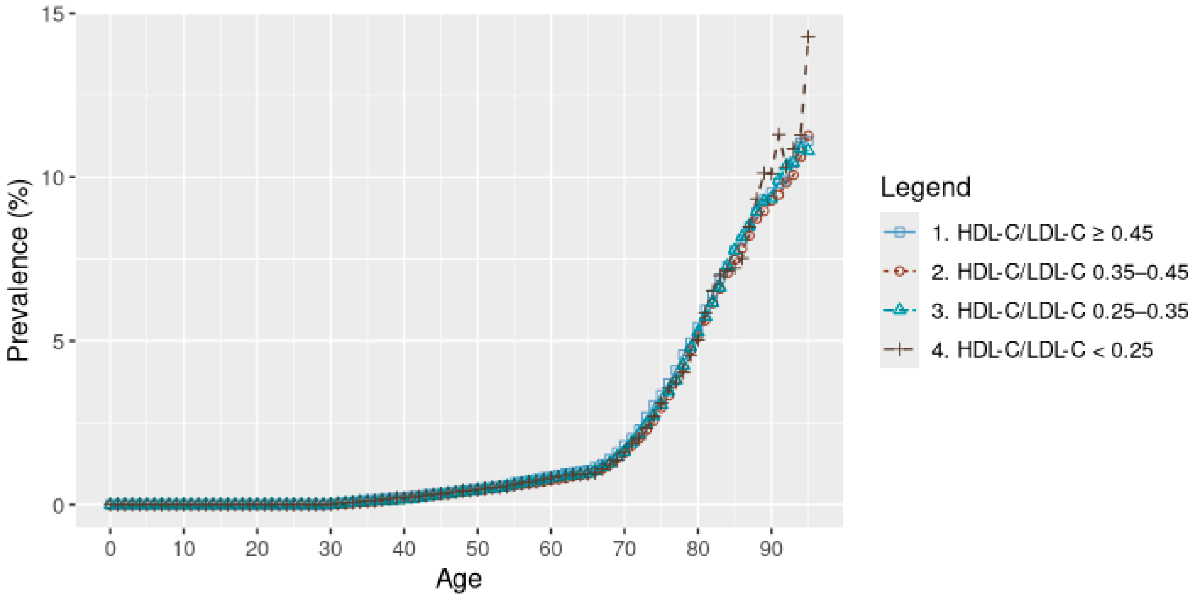

Figure S18. *Male Atrial Fibrilation Prevalence by Risk Group.*

Systolic Blood Pressure

```
hba1csbpTablev<-hba1csbpTable[hba1csbpTable$sex=="Female",]
hba1csbpTablev<-hba1csbpTablev[hba1csbpTablev$age<=95,]
hba1csbpTablev<-hba1csbpTablev[hba1csbpTablev$age>=18,]
hba1csbpTablev$Legend<-hba1csbpTablev$riskGroup

hba1csbpTablev$Legend<-gsub(">=","≥",hba1csbpTablev$Legend)
hba1csbpTablev$Legend<-gsub(" - ","\u2013",hba1csbpTablev$Legend)
multilinePlot(hba1csbpTablev,"","Age","Mean SBP",age,SBP,textSize=12)
```

The Figure S19 presents mean systolic blood pressure (SBP) across age groups in females stratified by HDL-C/LDL-C risk group. Mean SBP increases progressively with advancing age, rising from approximately 122 mmHg in early adulthood to around 135–136 mmHg in older age groups. Differences between risk groups are relatively modest throughout most of the age spectrum. However, females in the very high-risk group (HDL-C/LDL-C < 0.25) consistently demonstrate slightly lower mean SBP values compared with the other groups due to high mortality in that population group.

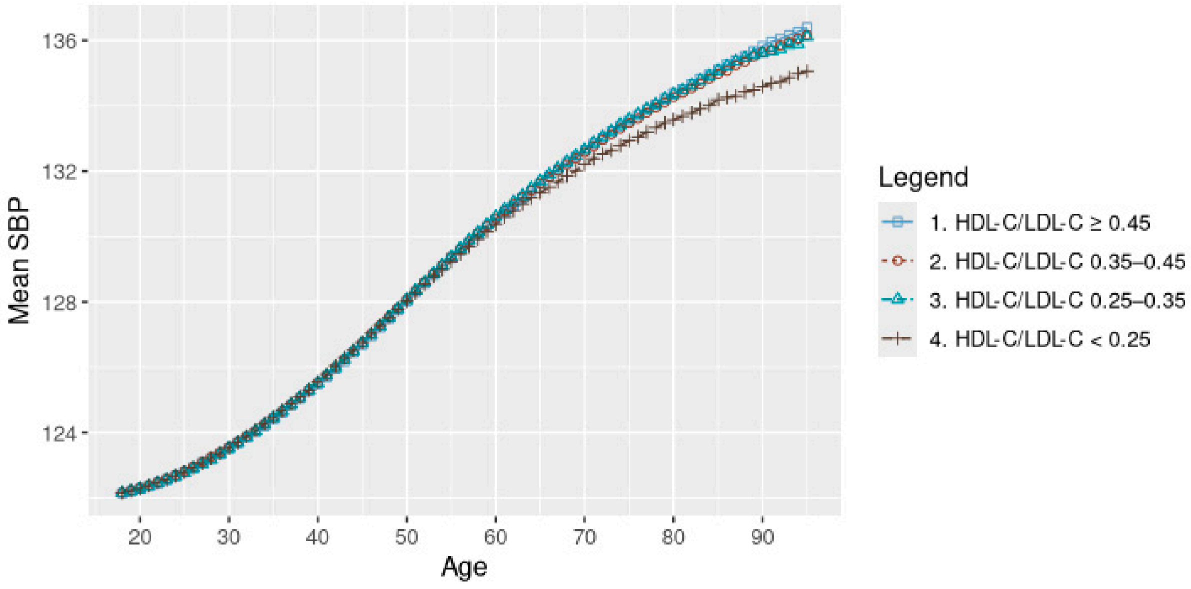

Figure S19. *Female SBP by Risk Group.*

```
hba1csbpTablev<-hba1csbpTable[hba1csbpTable$sex=="Male",]
hba1csbpTablev<-hba1csbpTablev[hba1csbpTablev$age<=95,]
hba1csbpTablev<-hba1csbpTablev[hba1csbpTablev$age>=18,]
hba1csbpTablev$Legend<-hba1csbpTablev$riskGroup

hba1csbpTablev$Legend<-gsub(">=","≥",hba1csbpTablev$Legend)
hba1csbpTablev$Legend<-gsub(" - ","\u2013",hba1csbpTablev$Legend)

multilinePlot(hba1csbpTablev,"","Age","Mean SBP",age,SBP,textSize=12)
```

The Figure S20 presents mean SBP across age groups in males stratified by HDL-C/LDL-C risk group. Mean SBP increases steadily with advancing age, rising from approximately 122 mmHg in early adulthood to nearly 136 mmHg in older age groups. Differences between risk groups are relatively small across most ages, however, males in the very high-risk group (HDL-C/LDL-C < 0.25) consistently exhibit lower mean SBP values compared with the other groups, particularly at older ages. A slight decline in SBP is also observed in the oldest individuals within the very high-risk group reflecting higher mortality in the very high risk group.

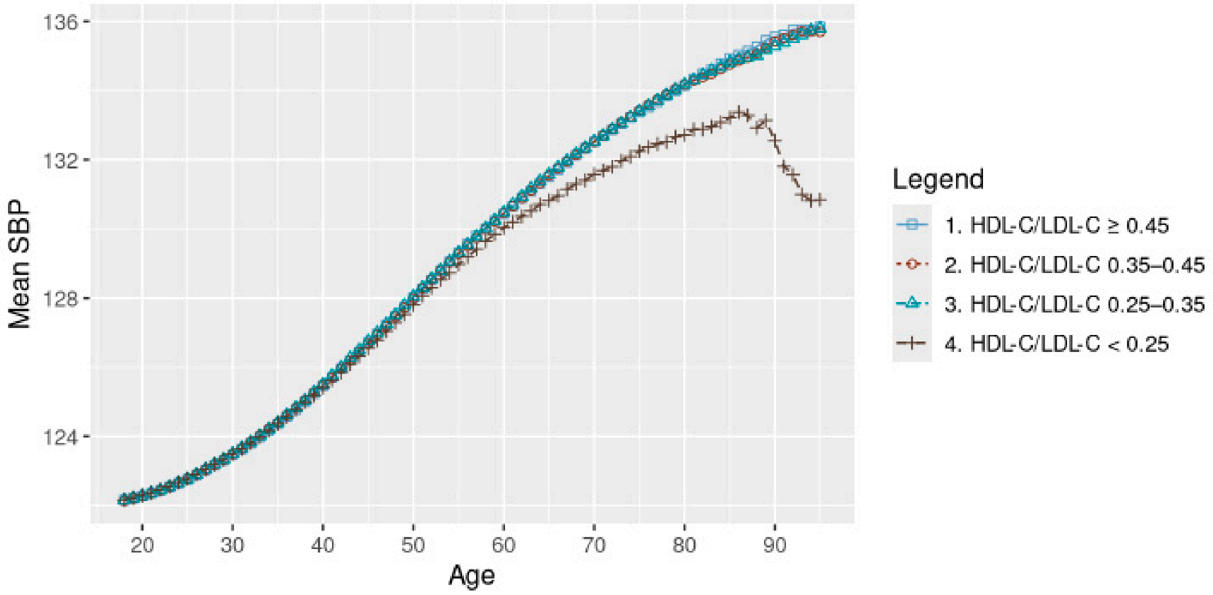

Figure S20. *Male SBP by Risk Group.*

Outcomes

Causes of Death

```
mortTable<-readRDS("rds/mortTable.RDS")

rgroups<-unique(mortTable$riskGroup)
mortTable1<-head(
  mortTable[mortTable$riskGroup==rgroups[[1]],] %>% arrange(desc(n))
  ,n=10)
mortTable2<-head(
  mortTable[mortTable$riskGroup==rgroups[[2]],] %>% arrange(desc(n))
  ,n=10)
mortTable3<-head(
  mortTable[mortTable$riskGroup==rgroups[[3]],] %>% arrange(desc(n))
  ,n=10)
mortTable4<-head(
  mortTable[mortTable$riskGroup==rgroups[[4]],] %>% arrange(desc(n))
  ,n=10)
mortTable<-rbind(mortTable4,mortTable3,mortTable2,mortTable1)
morteT<-pivot_wider(mortTable,id_cols = c(cod),
                names_from = c(riskGroup),
                values_from = c("n"))
teT<-morteT %>% mutate_if(is.numeric, format,big.mark=",",decimal.mark=".")
names(teT)[1]="Cause of Death"

ft <- flextable(teT)
ft <- set_caption(ft,caption="Top 10 causes of death by risk group")
ft<- padding(ft,padding =0,part="all")
ft <- fontsize(ft, size = 8,part="all")
ft <- autofit(ft)
ft
```

Table S4 presents the top ten causes of death across HDL-C/LDL-C risk groups. Ischaemic heart disease (I21) and other forms of heart disease (I22–I25) were the leading causes of death across all risk categories, with the highest numbers observed in the very high-risk group (HDL-C/LDL-C < 0.25). Respiratory diseases, including pneumonia (J18) and chronic lower respiratory diseases (J44), as well as malignant neoplasms such as lung cancer (C34), also contributed substantially to mortality across groups. Overall, the burden of cardiovascular mortality was markedly greater in higher-risk populations, whereas lower-risk groups showed relatively fewer deaths across most causes.

Table S4. *Top 10 causes of death by risk group.*

Cause of Death	4. HDL-C/LDL-C < 0.25	3. HDL-C/LDL-C 0.25 - 0.35	2. HDL-C/LDL-C 0.35 - 0.45	1. HDL-C/LDL-C ≥ 0.45
I21	37,459	14,759	6,207	2,445
I22-I25	28,389	7,642	4,160	3,664
E11	3,307	NA	NA	NA
C34	2,428	4,595	4,900	4,970
J18	1,846	6,106	7,658	8,366
I50	1,615	2,298	NA	NA
I60-I63	1,459	4,424	5,180	5,192

Cause of Death	4. HDL-C/LDL-C < 0.25	3. HDL-C/LDL-C 0.25 - 0.35	2. HDL-C/LDL-C 0.35 - 0.45	1. HDL-C/LDL-C ≥ 0.45
J44	1,445	4,021	4,802	5,101
F01-F99	1,282	3,672	4,502	4,860
C50	1,098	NA	NA	NA
R54	NA	2,695	3,475	3,848
I67	NA	2,307	2,743	3,035
C61	NA	NA	2,052	2,280

Quality of Life

```
eq5dTable<-readRDS("rds/eq5dTable.RDS")
geq5dTable<-eq5dTable[eq5dTable$sex=="Male",]
geq5dTable$Legend<-geq5dTable$riskGroup
multilinePlot(geq5dTable,"","Age","Total QALYs",age,EQ5D,textSize=12)
```

The Figure S21 presents age-specific QALY profiles in males stratified by HDL-C/LDL-C risk group. Total QALYs decline progressively with advancing age across all risk categories, with a markedly steeper reduction observed in higher-risk groups. Males in the very high-risk group (HDL-C/LDL-C < 0.25) demonstrate the most pronounced decline, with QALYs decreasing substantially from middle age onward and approaching zero at earlier ages compared with lower-risk groups. In contrast, lower-risk groups maintain higher QALY levels across the lifespan, indicating better overall survival and health-related quality of life..

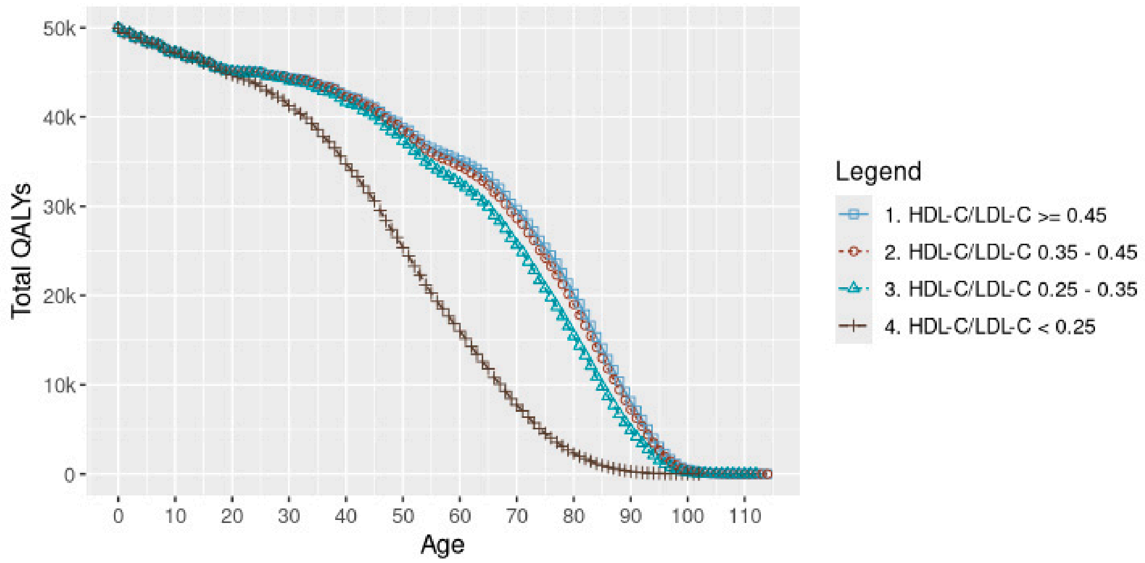

Figure S21. *Male QALY profiles.*

```
geq5dTable<-eq5dTable[eq5dTable$sex=="Female",]
geq5dTable$Legend<-geq5dTable$riskGroup
multilinePlot(geq5dTable,"","Age","Total QALYs",age,EQ5D,textSize=12)
```

The Figure S22 presents age-specific QALY profiles in females stratified by HDL-C/LDL-C risk group. Total QALYs decrease progressively with advancing age across all risk categories, with

*steeper declines observed in higher-risk groups. Females in the very high-risk group (HDL-C/LDL-C < 0.25) demonstrate the most pronounced reduction in QALYs, particularly from middle age onward, reaching near-zero values earlier than the lower-risk groups. In contrast, females in lower-risk categories maintain higher QALY levels across the lifespan, reflecting better overall survival and health-related quality of life outcomes***.**

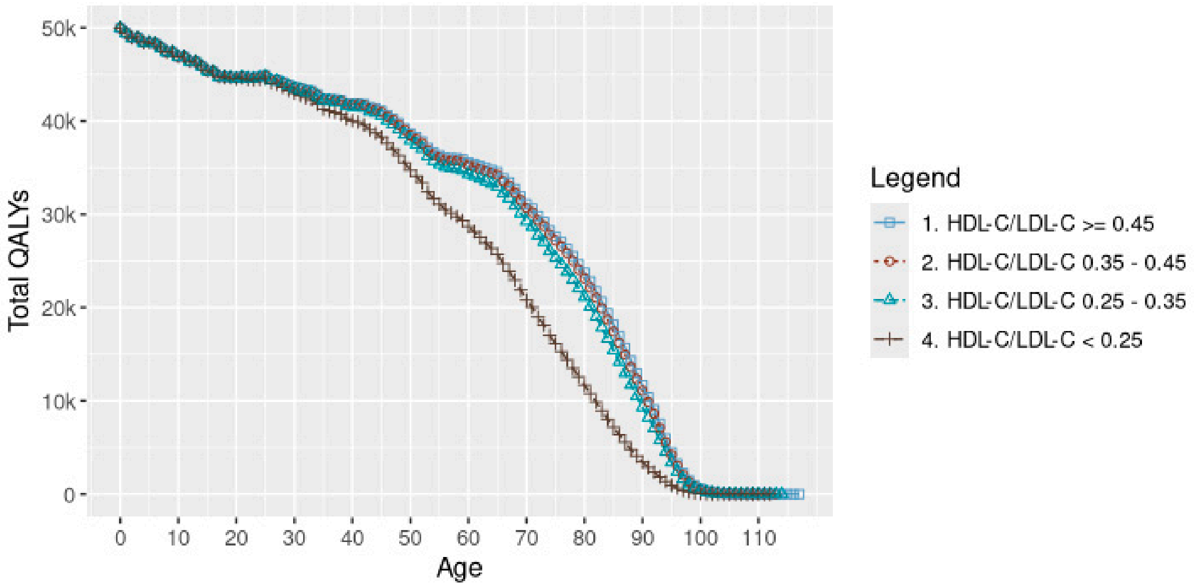

Figure S22. Female QALY profiles.
